# Supplementary material for: Mobilisation and analyses of publicly available SARS-CoV-2 data for pandemic responses
Source: Microb Genom. 2024 Feb 15;10(2):001188. doi: 10.1099/mgen.0.001188 (PMC10926692; doi:10.1099/mgen.0.001188)
Supplement: Supplementary material 1 [file mgen-10-1188-s001.pdf]

Pangolin Lineages for Submitted Sequences

| Pangolin lineage | Pangolin lineages counts |
|---|---|
| AY.4 | 640542 |
| BA.2 | 573487 |
| BA.1.1 | 562929 |
| B.1.1.7 | 542300 |
| AY.103 | 231711 |
| BA.1 | 231067 |
| AY.44 | 200932 |
| BA.2.12.1 | 143208 |
| AY.3 | 133027 |
| BA.1.17.2 | 124531 |
| AY.43 | 116421 |
| AY.25 | 109701 |
| BA.1.15 | 101258 |
| BA.2.9 | 100796 |
| AY.122 | 88760 |
| B.1.617.2 | 83981 |
| B.1.2 | 71043 |
| AY.4.2 | 69573 |
| AY.100 | 63567 |
| AY.25.1 | 58619 |
| BA.1.1.18 | 54603 |
| B.1 | 40661 |
| AY.39 | 39884 |
| AY.5 | 38747 |
| BA.5.2.1 | 38361 |
| BA.2.3 | 36370 |
| AY.98 | 33930 |
| B.1.526 | 33273 |
| BA.5.1 | 32830 |
| B.1.177 | 32816 |
| AY.47 | 32464 |
| AY.26 | 31954 |
| BA.1.15.1 | 30914 |
| AY.119 | 29183 |
| B.1.429 | 28898 |
| B.1.1 | 25938 |
| BA.1.18 | 24423 |
| AY.4.2.2 | 23846 |
| AY.120 | 23549 |
| AY.126 | 22752 |
| AY.6 | 22034 |
| BA.1.20 | 21667 |
| BA.2.10 | 20140 |
| AY.20 | 19199 |
| BA.5.5 | 19125 |
| BA.1.1.1 | 18802 |
| BA.4.1 | 17885 |
| BA.1.17 | 17876 |
| P.1 | 17538 |
| AY.118 | 17262 |
| BA.5.2 | 16979 |
| AY.75 | 16678 |

| | |
|---|---|
| AY.9.2 | 15396 |
| AY.46.6 | 14343 |
| AY.4.2.1 | 14288 |
| AY.3.1 | 13634 |
| B.1.160 | 13624 |
| AY.121 | 13586 |
| B.1.427 | 13264 |
| AY.98.1 | 13001 |
| B.1.1.519 | 12727 |
| AY.117 | 12399 |
| AY.4.6 | 11633 |
| BA.1.1.14 | 11618 |
| D.2 | 11453 |
| AY.46.5 | 11356 |
| AY.9 | 11309 |
| BA.2.23 | 11211 |
| BA.2.1 | 11086 |
| AY.14 | 11025 |
| B.1.637 | 10932 |
| AY.125 | 10836 |
| BA.1.1.15 | 10724 |
| AY.42 | 10389 |
| AY.54 | 10261 |
| BA.1.16 | 10124 |
| BA.4 | 9831 |
| AY.129 | 9601 |
| AY.127 | 9414 |
| AY.119.2 | 9182 |
| AY.7.1 | 8642 |
| AY.33 | 8546 |
| BA.5.6 | 8291 |
| B.1.243 | 7846 |
| BA.1.1.13 | 7256 |
| AY.113 | 7238 |
| B.1.351 | 7019 |
| AY.39.1 | 6534 |
| AY.111 | 6525 |
| AY.36 | 6443 |
| AY.43.6 | 6100 |
| BA.2.8 | 5907 |
| Q.3 | 5437 |
| BE.1 | 5204 |
| AY.34 | 5083 |
| AY.46 | 5014 |
| AY.13 | 4987 |
| BA.2.18 | 4824 |
| AY.39.1.1 | 4739 |
| AY.46.4 | 4702 |
| BA.5 | 4635 |
| BA.1.1.2 | 4562 |
| B | 4549 |
| BA.2.37 | 4395 |
| AY.114 | 4367 |
| AY.7 | 4360 |

| | |
|---|---|
| B.1.258 | 4198 |
| BD.1 | 4143 |
| B.1.621 | 4100 |
| BE.1.1 | 4052 |
| BA.4.6 | 4047 |
| AY.4.8 | 3823 |
| BA.2.7 | 3768 |
| AY.116.1 | 3695 |
| BA.1.1.12 | 3695 |
| B.1.234 | 3691 |
| AY.99.2 | 3662 |
| P.1.17 | 3530 |
| BA.2.12 | 3466 |
| BA.1.1.11 | 3454 |
| AY.34.1 | 3372 |
| B.1.1.222 | 3360 |
| AY.110 | 3305 |
| B.1.221 | 3295 |
| AY.4.2.3 | 3108 |
| B.1.177.4 | 3086 |
| B.1.1.37 | 3082 |
| AY.120.1 | 3027 |
| AY.107 | 3005 |
| P.1.10 | 2992 |
| R.1 | 2932 |
| AD.2 | 2904 |
| BA.2.36 | 2843 |
| AY.4.5 | 2828 |
| AY.43.4 | 2789 |
| BA.5.1.1 | 2634 |
| BA.1.14 | 2621 |
| B.1.177.45 | 2599 |
| B.1.240 | 2591 |
| AY.2 | 2583 |
| A.1 | 2523 |
| B.1.525 | 2502 |
| B.1.177.86 | 2450 |
| B.1.575 | 2426 |
| B.1.177.81 | 2313 |
| BE.3 | 2297 |
| B.1.177.57 | 2289 |
| BA.2.38 | 2263 |
| BA.2.10.1 | 2240 |
| BF.1 | 2233 |
| BA.1.19 | 2230 |
| BA.2.13 | 2200 |
| BA.1.12 | 2105 |
| Q.4 | 2105 |
| B.1.369 | 2094 |
| BF.5 | 2084 |
| B.1.311 | 1997 |
| AY.112 | 1985 |
| AY.1 | 1973 |
| AY.36.1 | 1932 |

| | |
|---|---|
| AY.5.3 | 1925 |
| XE | 1919 |
| B.1.623 | 1904 |
| AY.67 | 1856 |
| B.1.400 | 1848 |
| AY.37 | 1838 |
| B.1.595 | 1813 |
| BA.4.4 | 1811 |
| AY.121.1 | 1777 |
| AY.124 | 1775 |
| B.1.1.529 | 1772 |
| B.1.1.311 | 1673 |
| B.1.177.44 | 1671 |
| AY.4.9 | 1658 |
| AY.64 | 1627 |
| AY.23 | 1604 |
| AY.62 | 1584 |
| BA.1.15.2 | 1582 |
| BA.1.21 | 1576 |
| B.1.1.39 | 1555 |
| B.1.1.318 | 1551 |
| B.1.517 | 1541 |
| BA.1.1.4 | 1532 |
| AY.90 | 1521 |
| AY.25.3 | 1514 |
| BA.2.3.17 | 1508 |
| AY.4.3 | 1492 |
| BA.2.65 | 1456 |
| AY.48 | 1428 |
| AY.41 | 1421 |
| AY.4.10 | 1417 |
| BA.1.10 | 1397 |
| B.1.1.307 | 1388 |
| AY.57 | 1385 |
| AY.8 | 1365 |
| BA.2.14 | 1342 |
| AY.35 | 1323 |
| AY.4.15 | 1321 |
| AY.16 | 1317 |
| P.2 | 1295 |
| BA.2.22 | 1293 |
| AY.65 | 1288 |
| B.1.177.7 | 1261 |
| B.1.36.17 | 1261 |
| B.1.596 | 1260 |
| BA.4.2 | 1257 |
| B.1.1.317 | 1244 |
| B.1.561 | 1229 |
| C.37 | 1225 |
| AY.84 | 1219 |
| AY.46.1 | 1214 |
| AY.4.1 | 1213 |
| BA.1.1.16 | 1210 |
| B.1.1.1 | 1207 |

| | |
|---|---|
| B.1.258.17 | 1186 |
| AY.120.2.1 | 1183 |
| AY.4.4 | 1165 |
| XB | 1158 |
| BA.2.48 | 1138 |
| BA.5.2.3 | 1135 |
| AY.80 | 1130 |
| B.1.232 | 1106 |
| AY.122.3 | 1054 |
| B.1.609 | 1051 |
| B.1.1.291 | 1044 |
| BA.2.5 | 1027 |
| B.1.621.1 | 1025 |
| BA.2.26 | 1011 |
| B.1.177.16 | 1001 |
| BA.2.41 | 1000 |
| AY.70 | 990 |
| B.1.36 | 990 |
| AY.29 | 987 |
| BA.2.56 | 980 |
| BA.1.1.10 | 973 |
| BA.5.3.1 | 968 |
| AY.4.7 | 966 |
| BA.1.13 | 953 |
| AY.5.4 | 952 |
| A | 950 |
| B.1.1.70 | 942 |
| AY.52 | 934 |
| AY.46.2 | 926 |
| BA.2.47 | 896 |
| AY.51 | 887 |
| BA.1.9 | 877 |
| BA.5.1.3 | 877 |
| B.1.1.63 | 873 |
| B.1.110.3 | 871 |
| AY.10 | 870 |
| B.1.1.434 | 867 |
| C.36.3 | 866 |
| AY.106 | 860 |
| B.1.177.87 | 857 |
| B.1.241 | 856 |
| P.1.13 | 851 |
| B.1.1.369 | 850 |
| AY.116 | 848 |
| BA.2.69 | 841 |
| B.1.1.316 | 823 |
| B.1.177.17 | 814 |
| AY.68 | 811 |
| BA.2.21 | 807 |
| AY.105 | 801 |
| B.1.577 | 800 |
| B.40 | 800 |
| AY.43.8 | 798 |
| B.1.426 | 798 |

| | |
|---|---|
| BA.1.1.8 | 798 |
| B.1.1.416 | 797 |
| AY.87 | 791 |
| B.1.375 | 779 |
| A.2.5 | 778 |
| AY.134 | 777 |
| AY.45 | 776 |
| BA.1.5 | 766 |
| BA.2.50 | 764 |
| BA.2.23.1 | 762 |
| BA.4.1.1 | 756 |
| AY.4.14 | 753 |
| BA.2.31 | 753 |
| BA.5.3.3 | 746 |
| AY.101 | 738 |
| B.1.160.30 | 736 |
| AY.74 | 734 |
| AY.3.3 | 728 |
| BF.4 | 726 |
| AY.4.2.5 | 725 |
| B.1.565 | 720 |
| BA.5.1.2 | 718 |
| B.1.1.301 | 705 |
| B.1.177.10 | 699 |
| B.1.371 | 698 |
| B.1.177.54 | 686 |
| AY.38 | 685 |
| B.1.177.8 | 683 |
| BA.2.9.2 | 680 |
| B.1.177.56 | 676 |
| B.1.568 | 668 |
| B.1.367 | 664 |
| AY.92 | 662 |
| B.1.582 | 661 |
| AY.109 | 658 |
| B.1.509 | 654 |
| B.1.1.279 | 647 |
| B.1.588 | 642 |
| AY.7.2 | 639 |
| B.1.551 | 625 |
| B.1.1.372 | 623 |
| AY.11 | 618 |
| B.1.320 | 617 |
| B.1.177.62 | 610 |
| BA.2.9.1 | 595 |
| B.1.349 | 594 |
| B.1.160.16 | 584 |
| AY.32 | 583 |
| BA.1.7 | 581 |
| BA.2.9.3 | 577 |
| BG.2 | 577 |
| AY.60 | 571 |
| AY.98.1.1 | 570 |
| BA.2.6 | 568 |

| | |
|---|---|
| B.1.177.43 | 557 |
| AY.43.9 | 554 |
| B.1.617.1 | 551 |
| BA.2.32 | 548 |
| B.1.1.25 | 542 |
| BA.2.72 | 541 |
| BA.5.3 | 539 |
| AY.91 | 535 |
| B.1.389 | 535 |
| AY.132 | 534 |
| P.1.7 | 524 |
| B.1.126 | 519 |
| AY.81 | 516 |
| AZ.3 | 508 |
| B.1.139 | 508 |
| B.1.396 | 501 |
| BA.2.3.2 | 497 |
| BA.2.2 | 491 |
| B.1.239 | 489 |
| B.1.1.207 | 487 |
| BA.2.20 | 486 |
| B.1.564 | 485 |
| C.35 | 485 |
| B.1.1.198 | 484 |
| B.1.1.269 | 481 |
| B.1.1.51 | 481 |
| AY.43.3 | 478 |
| B.1.177.18 | 476 |
| B.1.36.27 | 471 |
| BA.2.39 | 466 |
| BA.2.16 | 460 |
| B.1.177.58 | 453 |
| BA.2.51 | 451 |
| B.1.1.348 | 449 |
| B.1.1.214 | 445 |
| AY.83 | 443 |
| AY.88 | 439 |
| B.1.409 | 439 |
| BA.1.8 | 437 |
| C.30 | 436 |
| B.1.1.432 | 431 |
| BA.2.19 | 430 |
| B.1.570 | 428 |
| B.1.361 | 426 |
| W.4 | 426 |
| AY.24 | 424 |
| XG | 423 |
| B.1.177.5 | 422 |
| B.1.177.20 | 419 |
| B.1.265 | 418 |
| AY.58 | 412 |
| BA.2.3.4 | 412 |
| AY.30 | 411 |
| AY.73 | 406 |

| | |
|---|---|
| Q.1 | 399 |
| B.1.404 | 398 |
| BA.2.52 | 398 |
| AY.108 | 397 |
| BF.2 | 389 |
| B.1.177.69 | 388 |
| BA.2.49 | 385 |
| BA.2.40.1 | 383 |
| B.1.346 | 379 |
| B.1.36.16 | 379 |
| B.1.324 | 378 |
| B.1.93 | 375 |
| B.1.305 | 374 |
| BA.2.76 | 372 |
| B.1.1.10 | 370 |
| B.1.351.3 | 368 |
| BA.1.1.3 | 366 |
| B.1.36.1 | 364 |
| B.1.503 | 364 |
| AY.33.2 | 357 |
| AY.122.4 | 353 |
| B.1.160.26 | 353 |
| B.1.177.15 | 353 |
| B.1.280 | 353 |
| AY.72 | 351 |
| B.1.391 | 347 |
| B.1.177.11 | 345 |
| AY.34.1.1 | 342 |
| AY.4.11 | 342 |
| AY.34.2 | 339 |
| B.1.36.8 | 335 |
| BA.5.1.4 | 331 |
| B.1.416.1 | 328 |
| B.3 | 328 |
| B.1.1.228 | 326 |
| BA.2.73 | 326 |
| B.1.177.19 | 325 |
| B.1.399 | 323 |
| AY.77 | 321 |
| B.1.111 | 320 |
| B.1.617 | 320 |
| C.17 | 320 |
| B.1.177.9 | 317 |
| B.33 | 315 |
| AY.53 | 314 |
| AY.71 | 311 |
| BA.1.1.7 | 311 |
| AY.39.2 | 307 |
| B.1.177.23 | 306 |
| P.1.1 | 306 |
| B.1.1.4 | 305 |
| BA.1.1.17 | 304 |
| A.23.1 | 302 |
| B.1.236 | 301 |

| | |
|---|---|
| BA.2.67 | 301 |
| AY.94 | 300 |
| B.1.403 | 300 |
| B.1.258.3 | 298 |
| BB.2 | 298 |
| B.28 | 297 |
| AY.85 | 295 |
| B.1.1.170 | 295 |
| B.1.1.253 | 294 |
| P.1.15 | 293 |
| B.1.384 | 292 |
| B.1.627 | 290 |
| P.1.14 | 286 |
| AY.3.4 | 285 |
| B.1.1.232 | 282 |
| AY.79 | 280 |
| B.1.235 | 280 |
| AY.4.13 | 277 |
| B.1.177.6 | 275 |
| B.1.289 | 275 |
| B.1.36.35 | 274 |
| C.16 | 274 |
| B.1.22 | 271 |
| B.1.1.44 | 269 |
| B.1.453 | 267 |
| BA.2.3.6 | 267 |
| B.1.576 | 266 |
| BA.2.3.15 | 265 |
| B.1.1.189 | 262 |
| B.1.1.464 | 262 |
| B.1.1.284 | 261 |
| B.1.177.75 | 261 |
| BA.5.3.2 | 259 |
| B.1.1.153 | 258 |
| B.1.619 | 256 |
| AY.122.6 | 254 |
| C.36 | 252 |
| B.1.1.286 | 249 |
| B.1.1.277 | 247 |
| B.1.177.55 | 246 |
| B.1.1.217 | 245 |
| B.1.1.303 | 244 |
| B.6 | 244 |
| B.1.221.1 | 243 |
| XN | 241 |
| B.1.8 | 239 |
| BA.2.3.12 | 237 |
| BA.2.25 | 236 |
| C.36.3.1 | 235 |
| BA.1.14.1 | 233 |
| B.1.1.255 | 232 |
| B.1.425 | 232 |
| B.1.1.192 | 230 |
| B.1.351.2 | 228 |

| | |
|---|---|
| B.1.471 | 228 |
| AY.56 | 227 |
| B.1.433 | 227 |
| BA.5.2.2 | 227 |
| AY.27 | 225 |
| AY.61 | 225 |
| B.1.177.77 | 224 |
| B.1.177.48 | 221 |
| B.1.516 | 221 |
| B.1.1.486 | 217 |
| B.1.1.83 | 217 |
| B.1.1.33 | 215 |
| B.1.1.74 | 214 |
| P.1.16 | 214 |
| AY.99.1 | 213 |
| A.3 | 212 |
| B.1.416 | 212 |
| B.23 | 212 |
| A.2.2 | 211 |
| BA.1.6 | 209 |
| BC.1 | 208 |
| B.1.1.385 | 207 |
| XM | 206 |
| B.1.1.294 | 205 |
| B.1.160.32 | 204 |
| AY.5.5 | 203 |
| B.1.1.297 | 202 |
| B.1.160.14 | 201 |
| B.1.1.171 | 200 |
| B.1.160.29 | 200 |
| B.1.298 | 198 |
| B.1.36.9 | 198 |
| B.1.13 | 195 |
| AY.43.1 | 194 |
| B.1.177.52 | 193 |
| B.1.1.186 | 191 |
| B.1.160.15 | 191 |
| BA.2.29 | 191 |
| B.1.1.241 | 190 |
| B.1.1.28 | 190 |
| B.1.160.7 | 189 |
| BF.1.1 | 189 |
| B.1.366 | 188 |
| B.1.610 | 188 |
| BA.2.3.13 | 187 |
| B.1.1.227 | 184 |
| B.1.1.420 | 184 |
| B.1.301 | 184 |
| AY.46.3 | 183 |
| B.1.332 | 183 |
| B.1.36.31 | 182 |
| AY.122.1 | 181 |
| AY.43.2 | 181 |
| B.1.206 | 180 |

| | |
|---|---|
| AY.91.1 | 179 |
| B.1.1.142 | 179 |
| B.1.1.26 | 179 |
| B.1.634 | 178 |
| B.1.36.29 | 177 |
| B.1.1.368 | 175 |
| BA.2.3.10 | 174 |
| B.1.177.60 | 173 |
| B.1.631 | 173 |
| B.1.443 | 172 |
| B.1.567 | 172 |
| B.1.1.304 | 171 |
| B.1.401 | 171 |
| B.1.413 | 170 |
| B.1.258.4 | 169 |
| BA.2.3.5 | 169 |
| B.1.1.67 | 168 |
| B.1.110 | 168 |
| BA.2.3.9 | 168 |
| B.1.9.4 | 167 |
| BA.2.71 | 167 |
| B.1.104 | 166 |
| AV.1 | 165 |
| AY.78 | 165 |
| B.1.160.11 | 165 |
| B.1.1.365 | 164 |
| B.1.356 | 164 |
| B.1.160.12 | 163 |
| B.1.112 | 162 |
| BA.2.45 | 162 |
| B.1.1.174 | 161 |
| B.1.599 | 159 |
| B.1.1.220 | 158 |
| AY.23.2 | 157 |
| B.1.549 | 157 |
| A.2 | 155 |
| B.1.1.231 | 155 |
| B.1.1.8 | 155 |
| B.1.1.523 | 154 |
| B.1.177.2 | 151 |
| B.1.177.26 | 151 |
| B.1.177.82 | 151 |
| B.1.597 | 151 |
| D.3 | 151 |
| AY.49 | 149 |
| B.1.229 | 149 |
| B.1.319 | 149 |
| BA.2.3.11 | 149 |
| AY.99 | 148 |
| B.1.492 | 148 |
| B.1.558 | 147 |
| B.1.1.306 | 146 |
| B.1.1.362 | 145 |
| B.1.336 | 143 |

| | |
|---|---|
| B.1.201 | 142 |
| B.1.625 | 142 |
| B.1.199 | 140 |
| BA.2.46 | 140 |
| L.3 | 140 |
| B.1.530 | 139 |
| W.3 | 139 |
| BA.2.55 | 138 |
| B.1.160.22 | 137 |
| B.1.362.2 | 137 |
| B.1.421 | 137 |
| BA.2.3.14 | 137 |
| XH | 137 |
| B.1.1.138 | 136 |
| B.1.620 | 136 |
| B.1.605 | 133 |
| AY.95 | 132 |
| AZ.2 | 132 |
| B.1.23 | 132 |
| B.1.377 | 131 |
| B.39 | 131 |
| BE.2 | 131 |
| B.1.221.2 | 130 |
| AY.124.1 | 129 |
| AY.43.7 | 129 |
| B.1.1.337 | 128 |
| B.1.160.10 | 128 |
| BA.2.74 | 127 |
| B.1.177.83 | 126 |
| B.1.258.7 | 126 |
| B.1.448 | 126 |
| B.11 | 125 |
| B.1.574 | 124 |
| C.12 | 124 |
| A.6 | 121 |
| B.1.3 | 121 |
| B.1.532 | 121 |
| P.1.12 | 121 |
| B.1.1.135 | 120 |
| B.1.1.240 | 120 |
| BA.2.34 | 120 |
| B.1.397 | 119 |
| B.1.160.31 | 118 |
| B.1.162 | 118 |
| B.1.337 | 115 |
| B.1.36.39 | 115 |
| B.1.523 | 115 |
| B.31 | 115 |
| AZ.2.1 | 114 |
| B.1.1.244 | 114 |
| B.1.160.20 | 114 |
| B.1.36.28 | 114 |
| B.1.124 | 113 |
| BA.2.64 | 113 |

| | |
|---|---|
| C.23 | 113 |
| B.1.177.65 | 112 |
| BA.2.3.1 | 112 |
| A.28 | 111 |
| B.1.580 | 111 |
| B.1.223 | 110 |
| B.1.340 | 110 |
| BA.2.44 | 110 |
| BA.2.75 | 110 |
| B.1.315 | 109 |
| AY.16.1 | 108 |
| B.1.260 | 108 |
| B.1.541 | 108 |
| B.4 | 108 |
| BA.1.21.1 | 108 |
| A.2.5.2 | 107 |
| B.1.1.200 | 107 |
| B.1.177.21 | 107 |
| B.1.177.32 | 107 |
| B.55 | 107 |
| B.1.36.2 | 106 |
| B.1.602 | 106 |
| BA.2.4 | 106 |
| BA.2.53 | 106 |
| P.1.17.1 | 106 |
| AY.123 | 105 |
| B.1.1.196 | 105 |
| B.1.1.274 | 105 |
| B.1.1.517 | 105 |
| B.1.166 | 105 |
| B.1.313 | 105 |
| B.1.338 | 105 |
| B.1.544 | 105 |
| B.1.626 | 105 |
| XL | 105 |
| AY.128 | 104 |
| B.1.1.141 | 104 |
| B.1.146 | 104 |
| B.1.408 | 104 |
| B.1.1.12 | 103 |
| B.1.1.305 | 103 |
| B.1.1.453 | 103 |
| B.1.177.51 | 103 |
| XZ | 103 |
| B.1.370 | 102 |
| BA.2.24 | 102 |
| BA.3 | 102 |
| B.1.1.225 | 101 |
| B.1.587 | 101 |
| BF.3 | 101 |
| C.3 | 101 |
| B.1.1.376 | 100 |
| B.1.1.47 | 100 |
| B.1.177.71 | 100 |

| | |
|---|---|
| B.1.469 | 100 |
| A.21 | 99 |
| B.1.1.115 | 99 |
| B.1.1.59 | 99 |
| B.1.560 | 99 |
| A.29 | 98 |
| AY.120.2 | 98 |
| B.1.1.161 | 98 |
| B.1.160.9 | 98 |
| B.1.268 | 96 |
| AY.15 | 95 |
| B.1.160.23 | 95 |
| B.1.612 | 95 |
| XQ | 95 |
| B.1.439 | 94 |
| BA.2.9.5 | 94 |
| C.37.1 | 94 |
| B.1.402 | 92 |
| B.1.225 | 91 |
| B.1.306 | 91 |
| B.1.630 | 91 |
| B.1.1.309 | 90 |
| B.1.1.50 | 90 |
| B.1.108 | 90 |
| B.29 | 90 |
| B.1.1.180 | 89 |
| B.1.1.219 | 89 |
| B.1.177.84 | 89 |
| BA.2.68 | 89 |
| AY.55 | 88 |
| B.1.1.205 | 88 |
| B.1.1.406 | 88 |
| AH.2 | 87 |
| B.1.210 | 87 |
| B.1.36.12 | 87 |
| B.1.533 | 87 |
| B.1.91 | 87 |
| B.1.1.3 | 86 |
| B.1.1.89 | 85 |
| B.1.273 | 85 |
| XY | 85 |
| B.1.190 | 84 |
| B.1.360 | 84 |
| AY.112.1 | 83 |
| AY.9.2.2 | 83 |
| B.1.314 | 83 |
| B.1.1.521 | 82 |
| B.1.398 | 82 |
| B.1.465 | 82 |
| BA.1.1.6 | 82 |
| B.1.1.204 | 81 |
| B.1.1.329 | 81 |
| B.1.451 | 81 |
| B.1.554 | 81 |

| | |
|---|---|
| B.1.1.216 | 80 |
| B.1.1.300 | 80 |
| B.1.177.53 | 80 |
| B.1.362 | 80 |
| B.1.435 | 80 |
| B.1.189 | 79 |
| B.1.480 | 79 |
| BA.2.42 | 79 |
| B.1.1.58 | 78 |
| B.1.105 | 78 |
| B.1.521 | 78 |
| B.1.636 | 78 |
| BA.2.54 | 77 |
| B.1.264 | 76 |
| B.1.603 | 76 |
| BA.2.17 | 76 |
| BA.2.59 | 76 |
| XW | 76 |
| B.1.1.128 | 75 |
| A.27 | 74 |
| AY.131 | 74 |
| B.1.177.28 | 74 |
| B.1.177.33 | 74 |
| XP | 74 |
| A.5 | 73 |
| B.1.423 | 73 |
| B.1.436 | 73 |
| B.1.524 | 73 |
| B.4.5 | 73 |
| BA.1.4 | 73 |
| B.1.1.125 | 72 |
| B.1.1.299 | 72 |
| B.1.385 | 72 |
| BA.2.10.3 | 72 |
| BA.2.25.1 | 72 |
| B.1.1.132 | 71 |
| B.1.160.28 | 71 |
| B.1.473 | 71 |
| P.1.2 | 71 |
| AY.86 | 70 |
| B.1.1.374 | 70 |
| BA.2.15 | 70 |
| N.5 | 70 |
| AA.1 | 69 |
| AY.119.1 | 69 |
| B.1.1.71 | 69 |
| B.1.36.24 | 69 |
| R.2 | 69 |
| B.1.117 | 68 |
| B.1.222 | 68 |
| B.1.381 | 68 |
| B.1.444 | 68 |
| B.1.485 | 68 |
| Z.1 | 68 |

| | |
|---|---|
| B.1.1.164 | 67 |
| B.1.1.270 | 67 |
| B.1.390 | 67 |
| B.1.452 | 67 |
| BA.2.62 | 67 |
| C.38 | 67 |
| B.1.1.61 | 66 |
| B.1.165 | 66 |
| B.1.363 | 66 |
| B.1.595.1 | 66 |
| B.45 | 66 |
| B.1.37 | 65 |
| B.1.382 | 65 |
| BA.2.28 | 65 |
| U.1 | 65 |
| B.1.1.322 | 64 |
| B.1.1.351 | 64 |
| B.1.258.10 | 64 |
| B.1.405 | 64 |
| B.1.515 | 64 |
| XAA | 64 |
| B.1.1.256 | 63 |
| BA.2.3.16 | 63 |
| BA.5.3.4 | 63 |
| B.1.550 | 62 |
| BA.2.63 | 62 |
| AY.124.1.1 | 61 |
| AY.25.1.1 | 61 |
| B.1.1.113 | 61 |
| B.1.1.308 | 61 |
| B.1.1.41 | 61 |
| BA.2.3.7 | 61 |
| XAC | 61 |
| AY.130 | 60 |
| B.1.1.236 | 60 |
| B.1.147 | 60 |
| B.1.177.73 | 60 |
| B.1.258.12 | 60 |
| B.3.1 | 60 |
| B.1.1.45 | 59 |
| B.1.113 | 59 |
| B.1.14 | 59 |
| B.1.1.518 | 58 |
| B.1.103 | 58 |
| B.1.258.5 | 58 |
| B.1.36.7 | 58 |
| B.1.632 | 58 |
| AY.123.1 | 57 |
| B.1.1.428 | 57 |
| B.1.344 | 57 |
| B.1.400.1 | 57 |
| B.1.438.4 | 57 |
| B.35 | 57 |
| B.1.379 | 56 |

| | |
|---|---|
| P.1.9 | 56 |
| B.1.78 | 55 |
| P.1.8 | 55 |
| AY.133 | 54 |
| B.1.1.165 | 54 |
| B.1.1.17 | 53 |
| B.1.238 | 53 |
| B.1.348 | 53 |
| B.1.355 | 53 |
| B.1.591 | 53 |
| B.1.1.384 | 52 |
| B.1.478 | 52 |
| AY.66 | 51 |
| B.1.1.130 | 51 |
| B.1.1.429 | 51 |
| B.1.282 | 51 |
| B.1.9.5 | 51 |
| B.1.1.450 | 50 |
| B.1.1.123 | 49 |
| B.1.1.419 | 49 |
| B.1.507 | 49 |
| BA.2.43 | 49 |
| B.1.1.218 | 48 |
| B.1.177.89 | 48 |
| B.1.258.14 | 48 |
| BA.1.14.2 | 48 |
| BA.2.27 | 48 |
| B.1.1.418 | 47 |
| B.1.119 | 47 |
| B.1.579 | 47 |
| B.46 | 47 |
| B.1.1.265 | 46 |
| B.1.1.397 | 46 |
| BA.1.15.3 | 46 |
| P.3 | 46 |
| AY.122.2 | 45 |
| AY.20.1 | 45 |
| B.1.1.344 | 45 |
| B.1.545 | 45 |
| B.20 | 45 |
| W.1 | 45 |
| B.1.1.285 | 44 |
| B.1.213 | 44 |
| B.1.251 | 44 |
| B.1.36.38 | 44 |
| B.1.637.1 | 44 |
| B.1.9 | 44 |
| B.1.1.158 | 43 |
| B.1.539 | 43 |
| BA.2.10.2 | 43 |
| XS | 43 |
| AY.59 | 42 |
| B.1.170 | 42 |
| B.1.218 | 42 |

| | |
|---|---|
| B.1.258.23 | 42 |
| B.1.563 | 42 |
| N.4 | 42 |
| B.1.1.251 | 41 |
| B.1.1.413 | 41 |
| B.1.1.487 | 41 |
| B.1.1.528 | 41 |
| B.1.214.2 | 41 |
| B.1.267 | 41 |
| B.1.429.1 | 41 |
| B.1.546 | 41 |
| B.1.575.1 | 41 |
| BA.2.78 | 41 |
| BA.4.7 | 41 |
| AY.112.2 | 40 |
| B.1.437 | 40 |
| BA.2.38.1 | 40 |
| BA.4.5 | 40 |
| AY.102 | 39 |
| B.1.1.290 | 39 |
| B.1.157 | 39 |
| XA | 39 |
| AY.21 | 38 |
| B.1.1.98 | 38 |
| B.1.177.85 | 38 |
| B.1.220 | 38 |
| B.1.258.6 | 38 |
| B.1.281 | 38 |
| B.1.499 | 38 |
| BA.1.1.9 | 38 |
| BA.5.2.4 | 38 |
| B.1.1.136 | 37 |
| B.1.1.15 | 37 |
| B.1.1.159 | 37 |
| B.1.1.239 | 37 |
| B.1.237 | 37 |
| B.61 | 37 |
| BA.2.35 | 37 |
| A.2.3 | 36 |
| B.1.177.31 | 36 |
| B.1.247 | 36 |
| B.1.441 | 36 |
| B.1.505 | 36 |
| B.43 | 36 |
| C.27 | 36 |
| XAF | 36 |
| AY.104 | 35 |
| B.1.1.168 | 35 |
| B.1.329 | 35 |
| B.1.552 | 35 |
| B.10 | 35 |
| BA.1.16.1 | 35 |
| BG.4 | 35 |
| C.2.1 | 35 |

| | |
|---|---|
| N.1 | 35 |
| N.8 | 35 |
| Q.7 | 35 |
| A.4 | 34 |
| AY.127.1 | 34 |
| B.1.1.139 | 34 |
| B.1.1.237 | 34 |
| B.1.274 | 34 |
| B.1.415 | 34 |
| B.1.501 | 34 |
| B.27 | 34 |
| B.58 | 34 |
| BA.2.33 | 34 |
| BA.4.1.4 | 34 |
| XAB | 34 |
| B.1.1.111 | 33 |
| B.1.1.229 | 33 |
| B.1.1.459 | 33 |
| B.1.287 | 33 |
| B.1.36.10 | 33 |
| B.1.420 | 33 |
| B.1.460 | 33 |
| B.1.474 | 33 |
| B.1.639 | 33 |
| B.1.96 | 33 |
| BA.2.79 | 33 |
| AY.5.2 | 32 |
| B.1.1.323 | 32 |
| B.1.1.97 | 32 |
| B.1.293 | 32 |
| B.1.466.1 | 32 |
| B.1.508 | 32 |
| B.1.81 | 32 |
| B.52 | 32 |
| B.57 | 32 |
| BA.2.2.1 | 32 |
| BA.2.30 | 32 |
| BA.3.1 | 32 |
| BB.1 | 32 |
| XF | 32 |
| A.2.5.1 | 31 |
| AY.4.2.4 | 31 |
| B.1.1.243 | 31 |
| B.1.177.63 | 31 |
| B.1.543 | 31 |
| AE.6 | 30 |
| AP.1 | 30 |
| AY.29.1 | 30 |
| B.1.1.144 | 30 |
| B.1.1.172 | 30 |
| B.1.1.48 | 30 |
| B.1.173 | 30 |
| B.1.188 | 30 |
| B.1.258.24 | 30 |

| | |
|---|---|
| B.1.556 | 30 |
| B.1.76 | 30 |
| B.1.97 | 30 |
| B.34 | 30 |
| BG.1 | 30 |
| AY.22 | 29 |
| B.1.1.120 | 29 |
| B.1.1.208 | 29 |
| B.1.1.315 | 29 |
| B.1.1.380 | 29 |
| B.1.1.480 | 29 |
| B.1.1.92 | 29 |
| B.1.328 | 29 |
| B.1.36.23 | 29 |
| B.1.38 | 29 |
| B.1.428.1 | 29 |
| B.59 | 29 |
| BA.2.11 | 29 |
| C.18 | 29 |
| AK.1 | 28 |
| AY.75.2 | 28 |
| B.1.1.213 | 28 |
| B.1.1.257 | 28 |
| B.1.1.512 | 28 |
| B.1.159 | 28 |
| B.1.177.72 | 28 |
| B.19 | 28 |
| B.26 | 28 |
| BA.2.58 | 28 |
| BA.4.3 | 28 |
| C.36.2 | 28 |
| XR | 28 |
| AS.2 | 27 |
| AY.39.3 | 27 |
| AY.42.1 | 27 |
| B.1.143 | 27 |
| B.1.160.19 | 27 |
| B.1.187 | 27 |
| B.1.302 | 27 |
| B.1.438.1 | 27 |
| B.1.445 | 27 |
| B.1.493 | 27 |
| B.1.498 | 27 |
| B.1.604 | 27 |
| B.1.640.1 | 27 |
| B.4.4 | 27 |
| B.5 | 27 |
| BA.2.60 | 27 |
| BA.2.66 | 27 |
| BA.2.9.4 | 27 |
| XV | 27 |
| AY.5.1 | 26 |
| B.1.1.258 | 26 |
| B.1.1.282 | 26 |

| | |
|---|---|
| B.1.12 | 26 |
| B.1.131 | 26 |
| B.1.442 | 26 |
| B.1.466.2 | 26 |
| B.30 | 26 |
| BA.2.70 | 26 |
| BA.2.81 | 26 |
| AF.1 | 25 |
| B.1.1.162 | 25 |
| B.1.1.57 | 25 |
| B.1.323 | 25 |
| B.1.330 | 25 |
| B.1.335 | 25 |
| B.1.36.34 | 25 |
| B.1.494 | 25 |
| XAE | 25 |
| XAG | 25 |
| B.1.1.109 | 24 |
| B.1.1.119 | 24 |
| B.1.1.445 | 24 |
| B.1.106 | 24 |
| B.1.294 | 24 |
| B.1.378 | 24 |
| B.1.40 | 24 |
| B.1.569 | 24 |
| B.47 | 24 |
| XAD | 24 |
| AN.1 | 23 |
| AY.29.2 | 23 |
| AY.33.1 | 23 |
| B.1.1.352 | 23 |
| B.1.177.3 | 23 |
| B.1.214.3 | 23 |
| B.1.231 | 23 |
| B.1.243.1 | 23 |
| B.1.596.1 | 23 |
| B.1.77 | 23 |
| BA.2.57 | 23 |
| BA.2.80 | 23 |
| C.14 | 23 |
| AY.28 | 22 |
| B.1.1.181 | 22 |
| B.1.250 | 22 |
| B.1.258.2 | 22 |
| B.1.304 | 22 |
| B.1.318 | 22 |
| B.1.407 | 22 |
| AY.82 | 21 |
| B.1.1.226 | 21 |
| B.1.1.382 | 21 |
| B.1.1.86 | 21 |
| B.1.177.30 | 21 |
| B.1.181 | 21 |
| B.1.438.3 | 21 |

| | |
|---|---|
| B.13 | 21 |
| BA.1.13.1 | 21 |
| A.23 | 20 |
| B.1.1.234 | 20 |
| B.1.1.355 | 20 |
| B.1.1.403 | 20 |
| B.1.1.426 | 20 |
| B.1.1.46 | 20 |
| B.1.128 | 20 |
| B.1.160.25 | 20 |
| B.1.160.33 | 20 |
| B.1.240.2 | 20 |
| B.1.245 | 20 |
| B.1.359 | 20 |
| B.1.415.1 | 20 |
| B.1.450 | 20 |
| B.1.566 | 20 |
| B.1.572 | 20 |
| B.1.621.2 | 20 |
| C.1.2 | 20 |
| N.7 | 20 |
| AY.17 | 19 |
| AY.50 | 19 |
| B.1.1.145 | 19 |
| B.1.1.34 | 19 |
| B.1.1.371 | 19 |
| B.1.1.482 | 19 |
| B.1.1.485 | 19 |
| B.1.1.77 | 19 |
| B.1.195 | 19 |
| B.1.578 | 19 |
| B.1.590 | 19 |
| B.1.594 | 19 |
| B.12 | 19 |
| B.51 | 19 |
| P.1.7.1 | 19 |
| AE.4 | 18 |
| B.1.1.203 | 18 |
| B.1.1.262 | 18 |
| B.1.1.266 | 18 |
| B.1.1.354 | 18 |
| B.1.1.361 | 18 |
| B.1.1.433 | 18 |
| B.1.1.437 | 18 |
| B.1.177.47 | 18 |
| B.1.325 | 18 |
| B.1.333 | 18 |
| B.1.438 | 18 |
| B.1.606 | 18 |
| B.1.635 | 18 |
| B.6.6 | 18 |
| BA.1.16.2 | 18 |
| N.6 | 18 |
| B.1.1.118 | 17 |

| | |
|---|---|
| B.1.1.133 | 17 |
| B.1.1.268 | 17 |
| B.1.1.40 | 17 |
| B.1.1.5 | 17 |
| B.1.215 | 17 |
| B.1.276 | 17 |
| B.1.383 | 17 |
| B.1.468 | 17 |
| B.1.557 | 17 |
| B.1.595.3 | 17 |
| B.1.88.1 | 17 |
| B.4.7 | 17 |
| BA.4.1.3 | 17 |
| B.1.1.101 | 16 |
| B.1.1.149 | 16 |
| B.1.1.176 | 16 |
| B.1.1.178 | 16 |
| B.1.1.254 | 16 |
| B.1.1.328 | 16 |
| B.1.1.378 | 16 |
| B.1.1.72 | 16 |
| B.1.177.35 | 16 |
| B.1.179 | 16 |
| B.1.254 | 16 |
| B.1.258.16 | 16 |
| B.1.310 | 16 |
| B.1.334 | 16 |
| B.1.341 | 16 |
| B.1.350 | 16 |
| B.1.393 | 16 |
| B.1.571 | 16 |
| B.1.595.4 | 16 |
| G.1 | 16 |
| XD | 16 |
| AE.7 | 15 |
| AS.1 | 15 |
| B.1.1.107 | 15 |
| B.1.1.13 | 15 |
| B.1.1.134 | 15 |
| B.1.1.210 | 15 |
| B.1.1.289 | 15 |
| B.1.1.320 | 15 |
| B.1.1.349 | 15 |
| B.1.1.463 | 15 |
| B.1.243.2 | 15 |
| B.1.36.18 | 15 |
| B.1.39 | 15 |
| B.1.428 | 15 |
| B.1.479 | 15 |
| B.1.589 | 15 |
| B.1.617.3 | 15 |
| BA.1.17.1 | 15 |
| BC.2 | 15 |
| C.26 | 15 |

| | |
|---|---|
| A.25 | 14 |
| AE.2 | 14 |
| AY.122.5 | 14 |
| AY.69 | 14 |
| AY.76 | 14 |
| B.1.1.166 | 14 |
| B.1.1.209 | 14 |
| B.1.1.302 | 14 |
| B.1.1.467 | 14 |
| B.1.1.507 | 14 |
| B.1.1.514 | 14 |
| B.1.1.55 | 14 |
| B.1.142 | 14 |
| B.1.163 | 14 |
| B.1.167 | 14 |
| B.1.184 | 14 |
| B.1.35 | 14 |
| B.1.36.22 | 14 |
| B.1.431 | 14 |
| B.1.466 | 14 |
| B.1.504 | 14 |
| B.1.586 | 14 |
| B.1.595.2 | 14 |
| BA.2.12.2 | 14 |
| BA.2.61 | 14 |
| C.31 | 14 |
| A.26 | 13 |
| AA.4 | 13 |
| B.1.1.147 | 13 |
| B.1.1.263 | 13 |
| B.1.1.312 | 13 |
| B.1.1.440 | 13 |
| B.1.1.441 | 13 |
| B.1.149 | 13 |
| B.1.160.13 | 13 |
| B.1.497 | 13 |
| BA.1.3 | 13 |
| L.2 | 13 |
| M.2 | 13 |
| N.2 | 13 |
| AA.7 | 12 |
| AA.8 | 12 |
| AY.25.1.2 | 12 |
| AY.63 | 12 |
| AZ.4 | 12 |
| B.1.1.155 | 12 |
| B.1.1.423 | 12 |
| B.1.1.461 | 12 |
| B.1.115 | 12 |
| B.1.198 | 12 |
| B.1.219 | 12 |
| B.1.428.2 | 12 |
| B.1.467 | 12 |
| B.1.531 | 12 |

| | |
|---|---|
| B.1.581 | 12 |
| B.1.640 | 12 |
| B.1.640.2 | 12 |
| C.1 | 12 |
| D.4 | 12 |
| P.7 | 12 |
| B.1.1.14 | 11 |
| B.1.1.187 | 11 |
| B.1.1.298 | 11 |
| B.1.1.341 | 11 |
| B.1.1.356 | 11 |
| B.1.1.404 | 11 |
| B.1.1.93 | 11 |
| B.1.1.95 | 11 |
| B.1.151 | 11 |
| B.1.168 | 11 |
| B.1.309 | 11 |
| B.1.321 | 11 |
| B.1.487 | 11 |
| B.1.527 | 11 |
| B.50 | 11 |
| BA.2.77 | 11 |
| BA.4.1.2 | 11 |
| BG.3 | 11 |
| C.20 | 11 |
| C.4 | 11 |
| M.3 | 11 |
| P.1.11 | 11 |
| P.1.4 | 11 |
| AA.6 | 10 |
| AY.43.5 | 10 |
| B.1.1.117 | 10 |
| B.1.1.148 | 10 |
| B.1.145 | 10 |
| B.1.178 | 10 |
| B.1.362.1 | 10 |
| B.1.406 | 10 |
| B.1.9.2 | 10 |
| BA.1.1.5 | 10 |
| C.5 | 10 |
| A.11 | 9 |
| B.1.1.169 | 9 |
| B.1.1.193 | 9 |
| B.1.1.242 | 9 |
| B.1.1.310 | 9 |
| B.1.1.347 | 9 |
| B.1.1.358 | 9 |
| B.1.1.359 | 9 |
| B.1.177.12 | 9 |
| B.1.177.50 | 9 |
| B.1.233 | 9 |
| B.1.249 | 9 |
| B.1.500 | 9 |
| B.1.502 | 9 |

| | |
|---|---|
| B.1.6 | 9 |
| B.1.629 | 9 |
| C.28 | 9 |
| Y.1 | 9 |
| AA.2 | 8 |
| AC.1 | 8 |
| AY.23.1 | 8 |
| AZ.5 | 8 |
| B.1.1.116 | 8 |
| B.1.1.163 | 8 |
| B.1.1.272 | 8 |
| B.1.1.280 | 8 |
| B.1.1.363 | 8 |
| B.1.1.402 | 8 |
| B.1.1.43 | 8 |
| B.1.1.458 | 8 |
| B.1.1.525 | 8 |
| B.1.164 | 8 |
| B.1.177.24 | 8 |
| B.1.177.74 | 8 |
| B.1.214 | 8 |
| B.1.258.11 | 8 |
| B.1.470 | 8 |
| B.1.495 | 8 |
| B.1.619.1 | 8 |
| B.32 | 8 |
| B.42 | 8 |
| BA.1.22 | 8 |
| N.3 | 8 |
| V.1 | 8 |
| AA.5 | 7 |
| AH.1 | 7 |
| AY.25.2 | 7 |
| AY.93 | 7 |
| B.1.1.175 | 7 |
| B.1.1.288 | 7 |
| B.1.1.327 | 7 |
| B.1.1.367 | 7 |
| B.1.1.370 | 7 |
| B.1.1.383 | 7 |
| B.1.1.389 | 7 |
| B.1.1.392 | 7 |
| B.1.1.409 | 7 |
| B.1.153 | 7 |
| B.1.205 | 7 |
| B.1.351.5 | 7 |
| B.1.36.20 | 7 |
| B.1.36.26 | 7 |
| B.1.491 | 7 |
| B.1.520 | 7 |
| B.1.585 | 7 |
| B.41 | 7 |
| C.33 | 7 |
| A.12 | 6 |

| | |
|---|---|
| A.2.5.3 | 6 |
| AT.1 | 6 |
| B.1.1.160 | 6 |
| B.1.1.177 | 6 |
| B.1.1.182 | 6 |
| B.1.1.185 | 6 |
| B.1.1.224 | 6 |
| B.1.1.296 | 6 |
| B.1.1.326 | 6 |
| B.1.1.421 | 6 |
| B.1.1.448 | 6 |
| B.1.1.54 | 6 |
| B.1.1.75 | 6 |
| B.1.110.2 | 6 |
| B.1.137 | 6 |
| B.1.211 | 6 |
| B.1.221.4 | 6 |
| B.1.256 | 6 |
| B.1.483 | 6 |
| B.1.548 | 6 |
| B.1.601 | 6 |
| A.7 | 5 |
| AH.3 | 5 |
| AK.2 | 5 |
| AY.103.1 | 5 |
| AY.125.1 | 5 |
| AY.4.17 | 5 |
| B.1.1.275 | 5 |
| B.1.1.338 | 5 |
| B.1.1.353 | 5 |
| B.1.1.395 | 5 |
| B.1.1.398 | 5 |
| B.1.182 | 5 |
| B.1.194 | 5 |
| B.1.208 | 5 |
| B.1.221.3 | 5 |
| B.1.258.22 | 5 |
| B.1.277 | 5 |
| B.1.395 | 5 |
| B.1.411 | 5 |
| B.1.424 | 5 |
| B.1.456 | 5 |
| B.1.462 | 5 |
| B.1.489 | 5 |
| B.1.510 | 5 |
| B.1.513 | 5 |
| B.1.518 | 5 |
| B.1.562 | 5 |
| B.1.600 | 5 |
| B.1.69 | 5 |
| B.1.9.1 | 5 |
| B.1.94 | 5 |
| B.37 | 5 |
| BA.2.3.8 | 5 |

| | |
|---|---|
| C.2 | 5 |
| C.30.1 | 5 |
| L.4 | 5 |
| P.1.12.1 | 5 |
| Q.2 | 5 |
| A.19 | 4 |
| AA.3 | 4 |
| AE.1 | 4 |
| AY.46.6.1 | 4 |
| B.1.1.230 | 4 |
| B.1.1.345 | 4 |
| B.1.1.373 | 4 |
| B.1.1.375 | 4 |
| B.1.1.38 | 4 |
| B.1.1.391 | 4 |
| B.1.161 | 4 |
| B.1.177.46 | 4 |
| B.1.263 | 4 |
| B.1.36.19 | 4 |
| B.1.418 | 4 |
| B.1.446 | 4 |
| B.1.540 | 4 |
| B.1.559 | 4 |
| B.18 | 4 |
| B.4.6 | 4 |
| BA.2.40 | 4 |
| C.13 | 4 |
| C.32 | 4 |
| N.9 | 4 |
| Q.6 | 4 |
| Q.8 | 4 |
| XAH | 4 |
| XJ | 4 |
| AG.1 | 3 |
| AY.19 | 3 |
| AY.24.1 | 3 |
| AY.26.1 | 3 |
| AY.31 | 3 |
| AY.39.1.2 | 3 |
| AY.4.12 | 3 |
| AY.4.16 | 3 |
| AY.5.6 | 3 |
| B.1.1.121 | 3 |
| B.1.1.129 | 3 |
| B.1.1.152 | 3 |
| B.1.1.194 | 3 |
| B.1.1.197 | 3 |
| B.1.1.27 | 3 |
| B.1.1.325 | 3 |
| B.1.1.331 | 3 |
| B.1.1.334 | 3 |
| B.1.1.335 | 3 |
| B.1.1.336 | 3 |
| B.1.1.364 | 3 |

| | |
|---|---|
| B.1.1.388 | 3 |
| B.1.1.408 | 3 |
| B.1.1.425 | 3 |
| B.1.1.431 | 3 |
| B.1.1.447 | 3 |
| B.1.1.500 | 3 |
| B.1.177.41 | 3 |
| B.1.177.76 | 3 |
| B.1.180 | 3 |
| B.1.203 | 3 |
| B.1.248 | 3 |
| B.1.252 | 3 |
| B.1.270 | 3 |
| B.1.432 | 3 |
| B.1.463 | 3 |
| B.1.496 | 3 |
| B.1.499.1 | 3 |
| B.1.555 | 3 |
| B.1.564.1 | 3 |
| B.1.618 | 3 |
| B.1.70 | 3 |
| BA.1.23 | 3 |
| BA.1.24 | 3 |
| D.5 | 3 |
| N.10 | 3 |
| P.6 | 3 |
| W.2 | 3 |
| A.24 | 2 |
| AB.1 | 2 |
| AM.3 | 2 |
| AY.3.2 | 2 |
| AY.75.3 | 2 |
| B.1.1.110 | 2 |
| B.1.1.201 | 2 |
| B.1.1.221 | 2 |
| B.1.1.271 | 2 |
| B.1.1.283 | 2 |
| B.1.1.319 | 2 |
| B.1.1.379 | 2 |
| B.1.1.381 | 2 |
| B.1.1.396 | 2 |
| B.1.1.410 | 2 |
| B.1.1.411 | 2 |
| B.1.1.424 | 2 |
| B.1.1.462 | 2 |
| B.1.1.506 | 2 |
| B.1.1.513 | 2 |
| B.1.1.524 | 2 |
| B.1.1.62 | 2 |
| B.1.1.88 | 2 |
| B.1.110.1 | 2 |
| B.1.116 | 2 |
| B.1.134 | 2 |
| B.1.177.14 | 2 |

| | |
|---|---|
| B.1.177.40 | 2 |
| B.1.177.59 | 2 |
| B.1.177.67 | 2 |
| B.1.192 | 2 |
| B.1.212 | 2 |
| B.1.224 | 2 |
| B.1.264.1 | 2 |
| B.1.284 | 2 |
| B.1.343 | 2 |
| B.1.36.33 | 2 |
| B.1.372 | 2 |
| B.1.428.3 | 2 |
| B.1.434 | 2 |
| B.1.459 | 2 |
| B.1.511 | 2 |
| B.1.535 | 2 |
| B.6.3 | 2 |
| BA.1.2 | 2 |
| BA.2.3.18 | 2 |
| C.25 | 2 |
| C.36.1 | 2 |
| P.1.3 | 2 |
| U.3 | 2 |
| XU | 2 |
| AE.5 | 1 |
| AE.8 | 1 |
| AJ.1 | 1 |
| AM.1 | 1 |
| AQ.1 | 1 |
| AW.1 | 1 |
| AY.112.3 | 1 |
| B.1.1.137 | 1 |
| B.1.1.154 | 1 |
| B.1.1.157 | 1 |
| B.1.1.16 | 1 |
| B.1.1.261 | 1 |
| B.1.1.332 | 1 |
| B.1.1.333 | 1 |
| B.1.1.339 | 1 |
| B.1.1.342 | 1 |
| B.1.1.357 | 1 |
| B.1.1.377 | 1 |
| B.1.1.393 | 1 |
| B.1.1.405 | 1 |
| B.1.1.407 | 1 |
| B.1.1.412 | 1 |
| B.1.1.417 | 1 |
| B.1.1.430 | 1 |
| B.1.1.449 | 1 |
| B.1.1.465 | 1 |
| B.1.1.515 | 1 |
| B.1.1.516 | 1 |
| B.1.1.526 | 1 |
| B.1.1.82 | 1 |

| | |
|---|---|
| B.1.1.99 | 1 |
| B.1.118 | 1 |
| B.1.127 | 1 |
| B.1.160.27 | 1 |
| B.1.160.8 | 1 |
| B.1.169 | 1 |
| B.1.177.38 | 1 |
| B.1.177.39 | 1 |
| B.1.177.42 | 1 |
| B.1.177.64 | 1 |
| B.1.177.68 | 1 |
| B.1.177.70 | 1 |
| B.1.258.15 | 1 |
| B.1.258.20 | 1 |
| B.1.258.9 | 1 |
| B.1.279 | 1 |
| B.1.350.1 | 1 |
| B.1.354 | 1 |
| B.1.357 | 1 |
| B.1.36.21 | 1 |
| B.1.36.36 | 1 |
| B.1.36.37 | 1 |
| B.1.422 | 1 |
| B.1.482 | 1 |
| B.1.506 | 1 |
| B.1.528 | 1 |
| B.1.537 | 1 |
| B.1.547 | 1 |
| B.1.593 | 1 |
| B.1.633 | 1 |
| B.15 | 1 |
| B.36 | 1 |
| B.38 | 1 |
| B.4.1 | 1 |
| B.4.8 | 1 |
| B.53 | 1 |
| B.6.8 | 1 |
| C.8 | 1 |
| K.2 | 1 |

Pangolin Lineages for the Systematic Analysis

| Pangolin lineage | Pangolin lineages counts |
|---|---|
| AY.4 | 223451 |
| BA.2 | 203004 |
| BA.1.1 | 181612 |
| B.1.1.7 | 138677 |
| AY.103 | 117046 |
| AY.44 | 98490 |
| BA.1 | 69993 |
| AY.3 | 64920 |
| AY.25 | 56184 |
| BA.2.12.1 | 45733 |
| B.1.617.2 | 40297 |
| BA.1.17.2 | 39511 |
| BA.1.15 | 36260 |
| AY.100 | 32511 |
| AY.25.1 | 31721 |
| AY.122 | 22239 |
| B.1.2 | 21804 |
| BA.5.2.1 | 21377 |
| AY.4.2 | 20101 |
| BA.1.1.18 | 19900 |
| BA.2.9 | 19508 |
| AY.43 | 18951 |
| B.1.177 | 18576 |
| AY.39 | 18537 |
| AY.5 | 17195 |
| B.1 | 16836 |
| AY.26 | 16528 |
| AY.119 | 15937 |
| AY.47 | 15431 |
| B.1.526 | 14840 |
| BA.2.3 | 14237 |
| B.1.1 | 12610 |
| AY.98 | 11088 |
| B.1.429 | 10913 |
| BA.5.1 | 10656 |
| AY.20 | 9691 |
| BA.5.2 | 9642 |
| AY.75 | 9165 |
| BA.1.15.1 | 8914 |
| AY.120 | 8887 |
| AY.6 | 8682 |
| AY.118 | 8351 |
| BA.5.5 | 8136 |
| AY.14 | 7537 |
| BA.1.18 | 7445 |
| AY.4.2.2 | 7420 |
| BA.2.10 | 7386 |
| BA.1.20 | 7320 |
| P.1 | 6639 |
| BA.4.1 | 6567 |
| AY.3.1 | 6482 |
| AY.117 | 5729 |

| | |
|---|---|
| B | 5588 |
| BA.1.1.2 | 5509 |
| AY.119.2 | 5450 |
| BA.2.23 | 5429 |
| BA.4.6 | 5288 |
| BA.2.1 | 5124 |
| B.1.427 | 5051 |
| AY.54 | 4869 |
| BA.5.6 | 4788 |
| BA.1.17 | 4596 |
| B.1.637 | 4573 |
| AY.4.2.1 | 4339 |
| AY.9.2 | 4289 |
| BA.1.1.15 | 4272 |
| B.1.1.519 | 4075 |
| BA.1.1.11 | 3951 |
| AY.113 | 3768 |
| AY.9 | 3693 |
| B.1.351 | 3564 |
| BA.1.1.1 | 3540 |
| AY.46.5 | 3452 |
| BA.2.8 | 3373 |
| BA.1.1.14 | 3368 |
| AY.13 | 3320 |
| AY.39.1 | 3130 |
| BA.1.16 | 3023 |
| B.1.243 | 2883 |
| AY.124 | 2605 |
| BQ.1.1 | 2549 |
| AY.46.4 | 2440 |
| BA.4 | 2434 |
| BF.10 | 2430 |
| BA.2.18 | 2424 |
| AY.98.1 | 2366 |
| BA.2.37 | 2276 |
| B.1.160 | 2271 |
| AY.116.1 | 2264 |
| AY.114 | 2250 |
| AY.126 | 2181 |
| AY.127 | 2132 |
| BA.1.1.13 | 2129 |
| BA.2.7 | 2105 |
| B.1.621 | 1986 |
| BE.1 | 1933 |
| B.1.1.37 | 1918 |
| B.1.1.369 | 1874 |
| AY.45 | 1862 |
| AY.111 | 1854 |
| AY.121 | 1826 |
| AY.125 | 1825 |
| BA.5.2.9 | 1796 |
| BA.2.38 | 1788 |
| BF.5 | 1723 |
| B.1.177.4 | 1686 |

| | |
|---|---|
| AD.2 | 1667 |
| AY.46.6 | 1650 |
| AY.7 | 1610 |
| AY.42 | 1593 |
| BF.26 | 1570 |
| B.1.177.57 | 1530 |
| BA.5.1.1 | 1510 |
| AY.36 | 1490 |
| BA.1.1.12 | 1487 |
| AY.110 | 1440 |
| BA.1.5 | 1439 |
| BE.1.1 | 1433 |
| AY.2 | 1418 |
| BQ.1 | 1388 |
| AY.37 | 1364 |
| BE.3 | 1340 |
| AY.5.3 | 1328 |
| BD.1 | 1307 |
| BA.2.9.5 | 1260 |
| B.1.221 | 1251 |
| AY.33 | 1250 |
| BA.2.10.1 | 1234 |
| B.1.234 | 1221 |
| AY.1 | 1220 |
| B.1.1.1 | 1217 |
| BA.2.9.2 | 1204 |
| AY.107 | 1191 |
| BF.7 | 1187 |
| AY.34 | 1180 |
| B.1.258 | 1176 |
| BA.5.1.30 | 1176 |
| B.40 | 1168 |
| P.1.17 | 1136 |
| AY.4.8 | 1132 |
| BA.5.2.20 | 1128 |
| AY.120.1 | 1110 |
| Q.3 | 1099 |
| P.1.10 | 1095 |
| B.1.575 | 1065 |
| B.1.1.222 | 1052 |
| XE | 1046 |
| AY.50 | 1039 |
| BA.2.56 | 1004 |
| BA.4.4 | 968 |
| BF.21 | 955 |
| BA.5.2.21 | 954 |
| AY.94 | 951 |
| BA.5.1.10 | 951 |
| BA.1.19 | 949 |
| BA.5.1.23 | 933 |
| AY.35 | 930 |
| BA.2.26 | 916 |
| BA.2.12 | 904 |
| A | 900 |

| | |
|---|---|
| BA.1.14 | 884 |
| B.1.1.318 | 877 |
| B.1.1.311 | 867 |
| AY.67 | 859 |
| AY.62 | 858 |
| AY.34.1 | 846 |
| AY.64 | 831 |
| B.1.36.17 | 818 |
| BF.28 | 818 |
| BF.27 | 810 |
| BA.2.27 | 798 |
| BA.5.1.22 | 792 |
| AY.4.3 | 784 |
| BA.2.22 | 776 |
| AY.46 | 754 |
| BA.5 | 749 |
| BA.2.5 | 748 |
| BA.1.9 | 745 |
| AY.129 | 735 |
| AY.53 | 735 |
| AY.46.1 | 727 |
| B.1.1.307 | 725 |
| BA.1.13 | 720 |
| BA.2.41 | 719 |
| R.1 | 714 |
| B.1.240 | 713 |
| B.1.177.16 | 711 |
| B.1.561 | 703 |
| B.1.623 | 702 |
| B.1.621.1 | 693 |
| AY.43.3 | 691 |
| AY.48 | 690 |
| BF.8 | 686 |
| B.1.525 | 676 |
| BA.2.29 | 636 |
| B.1.1.372 | 635 |
| AY.38 | 629 |
| B.1.22 | 629 |
| AY.4.5 | 624 |
| B.1.177.8 | 603 |
| BA.4.2 | 602 |
| B.1.1.176 | 590 |
| AY.90 | 588 |
| AY.25.3 | 584 |
| B.1.369 | 584 |
| P.2 | 583 |
| BF.1 | 579 |
| AY.52 | 576 |
| B.1.93 | 572 |
| BA.2.48 | 567 |
| B.1.177.54 | 566 |
| B.1.311 | 563 |
| BA.1.12 | 559 |
| C.37 | 551 |

| | |
|---|---|
| XB | 545 |
| A.1 | 533 |
| B.1.400 | 530 |
| AY.4.1 | 520 |
| BA.1.21 | 520 |
| BA.2.36 | 520 |
| B.1.36 | 515 |
| BA.1.15.2 | 514 |
| B.1.177.7 | 513 |
| BA.2.13 | 503 |
| AY.122.2 | 496 |
| B.1.391 | 493 |
| B.1.1.25 | 490 |
| AY.105 | 484 |
| B.1.1.434 | 483 |
| B.1.177.30 | 483 |
| BA.5.1.3 | 481 |
| B.1.595 | 478 |
| BA.5.1.25 | 476 |
| BA.2.31 | 475 |
| B.1.177.17 | 468 |
| B.1.177.81 | 467 |
| BA.1.1.16 | 466 |
| B.1.428 | 462 |
| AY.43.4 | 458 |
| BA.2.6 | 455 |
| B.1.1.301 | 452 |
| A.2.5 | 443 |
| BA.4.6.5 | 440 |
| AY.124.1 | 438 |
| AY.4.2.3 | 438 |
| B.1.139 | 438 |
| BA.2.65 | 438 |
| B.1.177.87 | 433 |
| B.1.1.28 | 428 |
| BA.5.1.2 | 427 |
| AY.116 | 420 |
| BA.1.7 | 416 |
| BA.1.1.4 | 409 |
| BA.2.34 | 409 |
| B.1.147 | 408 |
| BA.1.10 | 406 |
| B.33 | 401 |
| AY.8 | 397 |
| B.1.177.56 | 392 |
| B.1.8 | 392 |
| AY.43.8 | 391 |
| BA.1.1.10 | 390 |
| BA.2.21 | 382 |
| AY.88 | 381 |
| B.1.1.416 | 380 |
| AY.91 | 379 |
| B.1.241 | 379 |
| AY.74 | 378 |

| | |
|---|---|
| BA.5.8 | 375 |
| L.1 | 374 |
| BA.5.2.6 | 371 |
| AY.4.2.5 | 368 |
| BA.5.2.3 | 367 |
| BA.2.10.2 | 364 |
| B.3 | 357 |
| P.1.14 | 356 |
| B.1.596 | 354 |
| W.4 | 351 |
| B.1.389 | 350 |
| BA.2.3.17 | 350 |
| B.1.177.9 | 346 |
| BA.5.1.24 | 346 |
| B.1.617.1 | 341 |
| B.1.126 | 339 |
| B.1.177.18 | 339 |
| B.1.609 | 339 |
| B.1.232 | 336 |
| B.1.1.279 | 334 |
| A.2 | 333 |
| AY.112 | 329 |
| B.1.1.51 | 329 |
| AY.43.9 | 327 |
| BA.5.1.5 | 326 |
| BA.2.50 | 324 |
| B.1.91 | 322 |
| BE.1.4 | 320 |
| AZ.3 | 317 |
| B.1.177.5 | 316 |
| B.1.177.10 | 309 |
| BF.4 | 309 |
| B.1.177.19 | 305 |
| BF.13 | 304 |
| BF.11 | 300 |
| XBB.1 | 300 |
| B.1.177.60 | 298 |
| AY.3.3 | 297 |
| B.1.177.15 | 297 |
| B.1.177.69 | 294 |
| BQ.1.2 | 294 |
| B.1.1.10 | 293 |
| BA.5.1.6 | 291 |
| BQ.1.14 | 291 |
| AY.109 | 287 |
| B.1.320 | 283 |
| AY.16 | 280 |
| AY.87 | 280 |
| B.1.577 | 280 |
| BA.2.23.1 | 275 |
| AY.63 | 272 |
| AY.32 | 270 |
| AY.4.10 | 268 |
| BA.2.39 | 266 |

| | |
|---|---|
| XBB.1.5 | 265 |
| C.36.3 | 264 |
| AY.23 | 263 |
| B.1.438.1 | 263 |
| BA.5.3.1 | 263 |
| BF.7.4.1 | 262 |
| B.1.177.20 | 261 |
| B.1.1.333 | 259 |
| AY.4.9 | 258 |
| B.1.177.6 | 258 |
| B.1.551 | 257 |
| CH.1.1 | 256 |
| B.1.516 | 253 |
| B.1.1.142 | 252 |
| BA.2.16 | 252 |
| BA.2.32 | 250 |
| B.1.1.33 | 247 |
| BQ.1.3 | 247 |
| BA.4.1.1 | 245 |
| AY.22 | 244 |
| B.1.517 | 244 |
| AY.99.2 | 240 |
| B.1.177.32 | 240 |
| BF.7.4 | 240 |
| B.1.160.7 | 236 |
| BA.1.22 | 236 |
| B.1.1.157 | 235 |
| AZ.4 | 234 |
| BA.5.2.22 | 231 |
| BA.5.5.1 | 231 |
| AY.7.1 | 230 |
| BA.5.1.27 | 230 |
| BA.5.2.34 | 228 |
| BQ.1.1.3 | 227 |
| B.1.409 | 225 |
| B.1.1.312 | 224 |
| P.1.13 | 224 |
| AY.10 | 223 |
| BA.2.3.13 | 223 |
| C.16 | 221 |
| BA.5.1.18 | 220 |
| AY.121.1 | 219 |
| B.1.177.11 | 219 |
| BA.5.2.31 | 219 |
| AY.5.4 | 218 |
| B.1.177.52 | 217 |
| BA.4.1.6 | 215 |
| B.23 | 214 |
| AY.4.11 | 211 |
| B.1.396 | 211 |
| BA.2.13.1 | 211 |
| B.1.177.58 | 210 |
| BQ.1.11 | 208 |
| BQ.1.1.5 | 207 |

| | |
|---|---|
| B.1.110.3 | 205 |
| B.1.239 | 205 |
| AY.5.2 | 204 |
| BG.5 | 203 |
| BA.2.9.3 | 202 |
| BQ.1.12 | 202 |
| BA.1.1.5 | 200 |
| AY.83 | 199 |
| AY.70 | 198 |
| BA.1.1.17 | 198 |
| B.1.356 | 194 |
| BA.4.1.8 | 194 |
| B.1.177.72 | 193 |
| B.1.235 | 192 |
| B.1.565 | 192 |
| B.39 | 192 |
| AY.24 | 190 |
| AY.34.2 | 189 |
| B.1.1.317 | 189 |
| B.1.160.33 | 189 |
| BA.5.2.28 | 189 |
| AY.122.4 | 187 |
| B.1.588 | 187 |
| B.1.1.4 | 185 |
| BB.2 | 185 |
| B.1.503 | 183 |
| BG.2 | 183 |
| BQ.1.8 | 182 |
| AY.5.1 | 179 |
| B.1.1.198 | 178 |
| B.1.1.348 | 178 |
| B.1.36.22 | 177 |
| B.1.36.8 | 176 |
| B.1.426 | 175 |
| B.1.1.255 | 174 |
| BA.2.20 | 172 |
| B.1.1.253 | 171 |
| BA.2.67 | 171 |
| Q.4 | 171 |
| B.1.465 | 170 |
| BA.2.76 | 170 |
| C.30 | 169 |
| AY.68 | 168 |
| B.1.1.432 | 168 |
| B.1.222 | 168 |
| B.1.351.3 | 168 |
| AY.77 | 167 |
| BE.2 | 167 |
| AY.4.4 | 165 |
| B.1.1.464 | 165 |
| B.1.177.44 | 165 |
| B.1.1.70 | 164 |
| B.1.367 | 164 |
| BA.5.2.27 | 164 |

| | |
|---|---|
| B.1.1.220 | 163 |
| B.1.289 | 163 |
| B.1.582 | 163 |
| B.1.1.316 | 161 |
| BA.2.47 | 160 |
| BA.2.72 | 160 |
| B.1.1.217 | 159 |
| B.1.1.529 | 159 |
| AY.56 | 158 |
| B.28 | 156 |
| B.11 | 155 |
| BA.1.8 | 155 |
| BA.5.3.3 | 155 |
| B.1.13 | 154 |
| BA.1.1.8 | 154 |
| AY.11 | 151 |
| B.1.177.73 | 151 |
| B.1.223 | 151 |
| B.6 | 151 |
| BA.2.63 | 150 |
| AY.128 | 149 |
| AY.4.7 | 149 |
| BA.2.49 | 148 |
| BA.5.3 | 148 |
| B.1.258.3 | 147 |
| AY.124.1.1 | 146 |
| B.1.499 | 146 |
| B.1.568 | 146 |
| BA.5.1.12 | 146 |
| BQ.1.10 | 146 |
| BQ.1.15 | 146 |
| B.1.177.55 | 145 |
| B.1.36.27 | 145 |
| B.1.182 | 144 |
| BA.1.1.7 | 143 |
| A.23.1 | 142 |
| B.1.1.194 | 142 |
| BQ.1.1.4 | 142 |
| B.1.177.48 | 141 |
| BA.2.75.2 | 141 |
| BN.1.3 | 141 |
| B.1.177.82 | 140 |
| A.2.2 | 139 |
| B.1.36.9 | 139 |
| B.1.1.227 | 138 |
| B.1.1.83 | 138 |
| BA.5.5.2 | 138 |
| XN | 138 |
| B.1.104 | 137 |
| BA.2.9.1 | 137 |
| AY.106 | 136 |
| BA.2.3.2 | 136 |
| BF.39 | 136 |
| B.1.384 | 135 |

| | |
|---|---|
| BA.2.40.1 | 135 |
| BA.5.6.2 | 135 |
| AY.65 | 134 |
| BA.1.14.1 | 134 |
| AY.39.2 | 132 |
| AY.58 | 132 |
| B.1.1.44 | 132 |
| B.1.1.74 | 132 |
| B.59 | 132 |
| BA.5.9 | 132 |
| P.1.7 | 131 |
| B.1.505 | 130 |
| B.1.350 | 129 |
| BA.2.3.4 | 129 |
| C.8 | 129 |
| B.1.1.137 | 128 |
| B.55 | 128 |
| A.5 | 127 |
| AY.46.2 | 127 |
| BF.14 | 127 |
| BQ.1.13 | 127 |
| AY.27 | 126 |
| B.1.1.171 | 126 |
| B.1.111 | 126 |
| AY.17 | 124 |
| AY.60 | 124 |
| AY.81 | 123 |
| BA.5.2.23 | 122 |
| BE.4 | 122 |
| AE.8 | 121 |
| B.1.1.39 | 120 |
| BA.2.3.9 | 120 |
| B.1.380 | 119 |
| BQ.1.1.8 | 119 |
| BA.5.2.2 | 118 |
| P.1.15 | 118 |
| AY.7.2 | 117 |
| B.1.221.1 | 116 |
| B.1.627 | 116 |
| BF.25 | 115 |
| B.1.36.29 | 114 |
| B.1.399 | 114 |
| B.1.401 | 114 |
| BK.1 | 114 |
| BN.1.5 | 114 |
| BQ.1.1.18 | 114 |
| B.1.201 | 113 |
| B.1.36.16 | 113 |
| BQ.1.23 | 113 |
| B.1.1.41 | 112 |
| B.1.1.63 | 112 |
| B.1.471 | 112 |
| BA.5.1.35 | 112 |
| B.1.1.89 | 110 |

| | |
|---|---|
| BE.1.2 | 110 |
| AY.30 | 109 |
| B.1.258.2 | 109 |
| B.1.610 | 109 |
| BA.2.2 | 109 |
| BN.1 | 109 |
| B.1.1.207 | 108 |
| B.1.1.365 | 108 |
| B.1.36.21 | 107 |
| B.1.229 | 106 |
| B.29 | 106 |
| B.1.1.164 | 105 |
| B.1.153 | 105 |
| BQ.1.1.2 | 105 |
| B.1.1.303 | 104 |
| BQ.1.5 | 104 |
| B.1.338 | 103 |
| BA.5.1.7 | 103 |
| AY.122.1 | 102 |
| AY.41 | 102 |
| AY.92 | 102 |
| B.1.23 | 101 |
| BA.5.1.15 | 101 |
| BF.6 | 101 |
| B.61 | 100 |
| BA.2.3.10 | 100 |
| BA.2.52 | 100 |
| BE.1.1.1 | 100 |
| AY.120.2.1 | 99 |
| B.1.416.1 | 99 |
| B.1.631 | 99 |
| B.31 | 99 |
| C.3 | 98 |
| AY.108 | 97 |
| B.1.1.277 | 97 |
| B.1.1.286 | 97 |
| BQ.1.1.68 | 97 |
| BQ.1.6 | 97 |
| AY.51 | 96 |
| B.1.162 | 96 |
| BA.5.5.3 | 96 |
| BE.1.1.2 | 96 |
| C.35 | 96 |
| B.1.1.269 | 95 |
| B.1.280 | 95 |
| BA.2.3.6 | 95 |
| BA.5.2.16 | 95 |
| B.1.319 | 94 |
| BA.2.17 | 94 |
| BA.2.31.1 | 94 |
| BA.2.46 | 94 |
| AY.5.5 | 93 |
| B.1.1.228 | 93 |
| B.1.1.294 | 93 |

| | |
|---|---|
| B.1.1.8 | 93 |
| B.1.404 | 93 |
| B.1.617 | 93 |
| XJ | 93 |
| B.1.1.204 | 92 |
| B.1.112 | 92 |
| B.1.160.23 | 92 |
| B.1.1.240 | 91 |
| B.1.1.67 | 91 |
| B.1.177.85 | 91 |
| BA.2.73 | 91 |
| C.1.2 | 91 |
| B.1.177.47 | 90 |
| AZ.2 | 89 |
| B.45 | 88 |
| BQ.1.1.1 | 88 |
| B.1.190 | 86 |
| B.1.258.4 | 86 |
| B.1.36.24 | 86 |
| BA.2.3.15 | 86 |
| BF.32 | 86 |
| B.1.177.68 | 85 |
| B.1.268 | 85 |
| BA.5.2.19 | 85 |
| P.1.12 | 85 |
| BA.5.10 | 84 |
| B.1.1.153 | 83 |
| B.1.1.192 | 83 |
| B.1.1.200 | 83 |
| B.1.177.50 | 83 |
| B.1.523 | 83 |
| B.4 | 83 |
| BE.5 | 83 |
| D.2 | 83 |
| B.1.575.1 | 82 |
| BA.4.7 | 82 |
| B.1.1.59 | 81 |
| AS.1 | 80 |
| AY.4.6 | 80 |
| BA.2.3.5 | 80 |
| C.17 | 80 |
| B.1.1.170 | 79 |
| B.1.177.84 | 79 |
| B.52 | 79 |
| BF.3 | 79 |
| CN.2 | 79 |
| B.1.1.138 | 78 |
| B.1.1.237 | 78 |
| B.1.1.55 | 78 |
| B.1.14 | 78 |
| B.1.349 | 78 |
| BA.5.2.13 | 78 |
| BF.36 | 77 |
| CH.1.1.1 | 77 |

| | |
|---|---|
| B.1.36.18 | 76 |
| BQ.1.1.69 | 76 |
| AY.25.1.1 | 75 |
| B.1.1.121 | 75 |
| B.1.1.486 | 75 |
| B.1.39 | 75 |
| BA.5.3.5 | 75 |
| BF.1.1 | 75 |
| B.1.1.406 | 74 |
| BA.2.15 | 74 |
| AY.120.2 | 73 |
| AY.43.5 | 73 |
| B.1.1.189 | 73 |
| B.1.1.3 | 73 |
| B.1.634 | 73 |
| BA.5.2.35 | 73 |
| B.1.375 | 71 |
| AY.4.13 | 70 |
| B.1.1.209 | 70 |
| B.1.313 | 70 |
| BE.1.4.1 | 70 |
| BN.1.3.1 | 70 |
| B.1.1.296 | 69 |
| B.1.1.309 | 69 |
| B.1.177.77 | 69 |
| B.1.560 | 69 |
| BA.2.55 | 69 |
| BA.4.6.1 | 69 |
| XAS | 69 |
| AY.49 | 68 |
| AY.85 | 68 |
| B.1.1.208 | 68 |
| B.1.105 | 68 |
| B.1.179 | 68 |
| BF.16 | 68 |
| B.1.1.50 | 67 |
| B.1.258.7 | 67 |
| B.1.361 | 67 |
| B.1.558 | 67 |
| B.1.626 | 67 |
| BA.5.2.18 | 67 |
| B.1.1.300 | 66 |
| B.4.5 | 66 |
| BA.2.11 | 66 |
| BA.2.51 | 66 |
| BA.3 | 66 |
| A.2.3 | 65 |
| AH.3 | 65 |
| B.1.1.125 | 65 |
| B.1.1.71 | 65 |
| B.1.177.86 | 65 |
| BA.5.2.8 | 65 |
| B.1.370 | 64 |
| B.1.443 | 64 |

| | |
|---|---|
| B.1.532 | 64 |
| B.1.78 | 64 |
| BA.2.3.20 | 64 |
| BQ.1.1.22 | 64 |
| CK.1 | 64 |
| B.1.336 | 63 |
| B.1.599 | 63 |
| B.1.258.5 | 62 |
| B.1.36.28 | 62 |
| BA.1.6 | 62 |
| BA.2.25 | 62 |
| BA.2.9.7 | 62 |
| BA.4.1.9 | 62 |
| BM.1.1.3 | 62 |
| A.3 | 61 |
| AY.99 | 61 |
| B.1.1.368 | 61 |
| B.1.36.7 | 61 |
| AY.43.2 | 60 |
| B.1.1.297 | 60 |
| B.1.301 | 60 |
| BA.2.24 | 60 |
| B.1.220 | 59 |
| B.1.3 | 59 |
| BA.2.35 | 59 |
| BA.2.81 | 59 |
| BA.5.1.21 | 59 |
| BA.5.2.26 | 59 |
| AY.66 | 58 |
| B.1.1.299 | 58 |
| BA.2.75 | 58 |
| Y.1 | 58 |
| B.1.265 | 57 |
| BA.5.2.57 | 57 |
| B.1.1.115 | 56 |
| B.1.1.270 | 56 |
| B.1.1.282 | 56 |
| B.1.579 | 56 |
| BA.2.14 | 56 |
| BA.2.3.14 | 56 |
| BA.5.2.33 | 56 |
| BF.7.5 | 56 |
| XAZ | 56 |
| B.1.177.2 | 55 |
| BA.2.2.1 | 55 |
| BN.1.4 | 55 |
| B.1.1.274 | 54 |
| B.1.258.17 | 54 |
| B.1.408 | 54 |
| BE.1.2.1 | 54 |
| L.2 | 54 |
| AY.102 | 53 |
| B.1.157 | 53 |
| B.1.351.5 | 53 |

| | |
|---|---|
| B.1.36.1 | 53 |
| B.1.36.35 | 53 |
| BE.7 | 53 |
| N.5 | 53 |
| B.1.1.54 | 52 |
| B.1.177.53 | 52 |
| B.1.602 | 52 |
| BA.2.75.1 | 52 |
| XBB.2 | 52 |
| AY.84 | 51 |
| B.1.1.14 | 51 |
| B.1.1.232 | 51 |
| B.1.324 | 51 |
| BU.1 | 51 |
| Q.1 | 51 |
| B.1.166 | 50 |
| B.1.422 | 50 |
| B.1.625 | 50 |
| BQ.1.1.7 | 50 |
| CM.2 | 50 |
| XQ | 50 |
| A.4 | 49 |
| B.1.250 | 49 |
| B.1.298 | 49 |
| B.1.346 | 49 |
| B.1.509 | 49 |
| B.26 | 49 |
| XZ | 49 |
| B.1.1.289 | 48 |
| B.1.36.31 | 48 |
| B.1.362.2 | 48 |
| BA.2.57 | 48 |
| BA.5.1.9 | 48 |
| BE.1.4.2 | 48 |
| BQ.1.1.6 | 48 |
| B.1.1.12 | 47 |
| B.1.1.196 | 47 |
| B.1.1.5 | 47 |
| B.1.238 | 47 |
| B.1.258.12 | 47 |
| B.1.36.36 | 47 |
| BA.2.4 | 47 |
| AP.1 | 46 |
| AY.15 | 46 |
| B.1.1.149 | 46 |
| B.1.1.420 | 46 |
| B.1.177.65 | 46 |
| BA.2.64 | 46 |
| BF.2 | 46 |
| B.1.1.305 | 45 |
| B.1.137 | 45 |
| B.1.36.39 | 45 |
| B.1.40 | 45 |
| B.1.480 | 45 |

| | |
|---|---|
| B.1.576 | 45 |
| B.15 | 45 |
| BA.5.1.4 | 45 |
| BA.5.2.53 | 45 |
| XL | 45 |
| B.1.1.234 | 44 |
| ED.2 | 44 |
| XP | 44 |
| AY.86 | 43 |
| B.1.1.161 | 43 |
| B.1.1.216 | 43 |
| B.1.1.256 | 43 |
| B.1.1.306 | 43 |
| B.1.1.408 | 43 |
| B.1.1.45 | 43 |
| B.1.117 | 43 |
| B.1.164 | 43 |
| B.1.36.12 | 43 |
| B.1.600 | 43 |
| B.1.96 | 43 |
| BA.2.25.1 | 43 |
| BA.2.62 | 43 |
| BA.2.68 | 43 |
| BA.2.75.5 | 43 |
| XBE | 43 |
| B.1.1.174 | 42 |
| B.1.1.181 | 42 |
| B.1.160.32 | 42 |
| B.1.556 | 42 |
| B.1.630 | 42 |
| BA.2.44 | 42 |
| BQ.1.1.41 | 42 |
| C.27 | 42 |
| DE.1 | 42 |
| AZ.2.1 | 41 |
| B.1.1.123 | 41 |
| B.1.1.394 | 41 |
| B.1.177.14 | 41 |
| B.1.210 | 41 |
| B.1.332 | 41 |
| B.4.6 | 41 |
| BF.9 | 41 |
| BM.4.1.1 | 41 |
| BN.1.2 | 41 |
| BQ.1.1.10 | 41 |
| CQ.2 | 41 |
| B.1.1.241 | 40 |
| B.1.1.326 | 40 |
| B.1.177.40 | 40 |
| B.35 | 40 |
| AY.73 | 39 |
| B.1.1.236 | 39 |
| B.1.165 | 39 |
| B.1.76 | 39 |

| | |
|---|---|
| BA.2.10.3 | 39 |
| BA.5.1.17 | 39 |
| BY.1 | 39 |
| AY.101 | 38 |
| AY.95 | 38 |
| B.1.1.251 | 38 |
| B.1.1.485 | 38 |
| B.1.177.43 | 38 |
| BF.41 | 38 |
| BF.7.21 | 38 |
| BF.7.7 | 38 |
| BL.1 | 38 |
| CP.1 | 38 |
| AY.79 | 37 |
| B.1.1.523 | 37 |
| B.1.177.26 | 37 |
| B.1.225 | 37 |
| B.1.264 | 37 |
| B.1.453 | 37 |
| B.27 | 37 |
| BA.5.3.2 | 37 |
| BB.1 | 37 |
| BQ.1.1.24 | 37 |
| A.2.5.2 | 36 |
| AW.1 | 36 |
| AY.19 | 36 |
| B.1.1.257 | 36 |
| B.1.1.362 | 36 |
| B.1.177.62 | 36 |
| B.1.251 | 36 |
| B.1.254 | 36 |
| B.1.282 | 36 |
| B.1.397 | 36 |
| B.1.445 | 36 |
| BA.1.1.3 | 36 |
| BA.1.1.6 | 36 |
| BA.2.3.12 | 36 |
| BQ.1.1.15 | 36 |
| BR.1 | 36 |
| XBB | 36 |
| A.21 | 35 |
| B.1.1.117 | 35 |
| B.1.1.128 | 35 |
| B.1.1.213 | 35 |
| B.1.1.374 | 35 |
| B.1.398 | 35 |
| B.10 | 35 |
| B.57 | 35 |
| BA.5.1.36 | 35 |
| C.37.1 | 35 |
| EF.1.1 | 35 |
| W.3 | 35 |
| A.28 | 34 |
| B.1.1.154 | 34 |

| | |
|---|---|
| B.1.1.351 | 34 |
| B.1.416 | 34 |
| B.1.620 | 34 |
| B.46 | 34 |
| BA.2.45 | 34 |
| BA.2.59 | 34 |
| BA.2.74 | 34 |
| BA.5.2.7 | 34 |
| BF.7.6 | 34 |
| B.1.1.168 | 33 |
| B.1.1.517 | 33 |
| B.1.168 | 33 |
| B.1.9 | 33 |
| B.3.1 | 33 |
| B.58 | 33 |
| BA.4.6.4 | 33 |
| CA.5 | 33 |
| CK.2.1 | 33 |
| AY.39.3 | 32 |
| B.1.1.134 | 32 |
| B.1.1.219 | 32 |
| B.1.1.262 | 32 |
| B.1.236 | 32 |
| B.1.242 | 32 |
| B.1.362 | 32 |
| B.1.546 | 32 |
| B.1.548 | 32 |
| B.1.632 | 32 |
| BA.2.71 | 32 |
| BF.15 | 32 |
| BQ.1.1.52 | 32 |
| BQ.1.13.1 | 32 |
| C.14 | 32 |
| EF.2 | 32 |
| P.1.16 | 32 |
| AY.57 | 31 |
| B.1.110 | 31 |
| B.1.160.22 | 31 |
| B.1.177.33 | 31 |
| B.1.533 | 31 |
| B.20 | 31 |
| BA.2.33 | 31 |
| BA.5.11 | 31 |
| BQ.1.1.51 | 31 |
| BQ.1.8.2 | 31 |
| C.38 | 31 |
| DN.1.1 | 31 |
| K.3 | 31 |
| B.1.1.304 | 30 |
| B.1.597 | 30 |
| BA.1.21.1 | 30 |
| BA.1.4 | 30 |
| BA.5.2.59 | 30 |
| BE.1.3 | 30 |

| | |
|---|---|
| BN.1.7 | 30 |
| BQ.1.1.32 | 30 |
| BQ.1.10.1 | 30 |
| CA.1 | 30 |
| P.1.1 | 30 |
| XBB.1.2 | 30 |
| AY.122.6 | 29 |
| AY.55 | 29 |
| B.1.1.329 | 29 |
| B.1.1.518 | 29 |
| B.1.304 | 29 |
| B.1.305 | 29 |
| B.1.314 | 29 |
| B.1.379 | 29 |
| B.1.405 | 29 |
| B.34 | 29 |
| BA.5.1.19 | 29 |
| BQ.1.16 | 29 |
| Z.1 | 29 |
| AL.1 | 28 |
| AV.1 | 28 |
| AY.112.2 | 28 |
| AY.71 | 28 |
| B.1.1.291 | 28 |
| B.1.1.93 | 28 |
| B.1.140 | 28 |
| B.1.177.67 | 28 |
| B.1.276 | 28 |
| B.1.337 | 28 |
| B.1.36.20 | 28 |
| B.1.366 | 28 |
| B.1.378 | 28 |
| B.1.607 | 28 |
| B.1.9.1 | 28 |
| BA.3.1 | 28 |
| EF.1 | 28 |
| XW | 28 |
| A.2.5.1 | 27 |
| AS.2 | 27 |
| B.1.1.267 | 27 |
| B.1.1.337 | 27 |
| B.1.1.43 | 27 |
| B.1.1.61 | 27 |
| B.1.160.26 | 27 |
| B.1.177.80 | 27 |
| B.1.413 | 27 |
| B.1.439 | 27 |
| BA.5.2.25 | 27 |
| BA.5.6.1 | 27 |
| EB.1 | 27 |
| B.1.1.197 | 26 |
| B.1.1.273 | 26 |
| B.1.1.95 | 26 |
| B.1.435 | 26 |

| | |
|---|---|
| BA.1.1.9 | 26 |
| BA.1.15.3 | 26 |
| BA.5.1.33 | 26 |
| BF.31 | 26 |
| BM.1.1 | 26 |
| BQ.1.1.23 | 26 |
| CF.1 | 26 |
| XM | 26 |
| AY.39.1.2 | 25 |
| B.1.1.98 | 25 |
| B.1.177.83 | 25 |
| B.1.36.23 | 25 |
| B.1.403 | 25 |
| B.1.598 | 25 |
| B.1.605 | 25 |
| B.19 | 25 |
| B.6.6 | 25 |
| BA.2.78 | 25 |
| BF.11.1 | 25 |
| BQ.1.1.31 | 25 |
| EE.1 | 25 |
| XBB.3.2 | 25 |
| AK.1 | 24 |
| AY.119.1 | 24 |
| AY.4.2.4 | 24 |
| AY.72 | 24 |
| B.1.160.30 | 24 |
| B.1.177.51 | 24 |
| B.1.415.1 | 24 |
| B.1.418 | 24 |
| B.1.433 | 24 |
| B.1.469 | 24 |
| B.1.515 | 24 |
| B.1.97 | 24 |
| BA.2.42 | 24 |
| BA.2.53 | 24 |
| BA.5.2.24 | 24 |
| BA.5.2.44 | 24 |
| BA.5.2.54 | 24 |
| BF.40 | 24 |
| BQ.1.1.19 | 24 |
| BQ.1.1.27 | 24 |
| C.1 | 24 |
| DC.1 | 24 |
| B.1.1.186 | 23 |
| B.1.1.231 | 23 |
| B.1.1.448 | 23 |
| B.1.402 | 23 |
| B.1.438.4 | 23 |
| B.1.460 | 23 |
| B.1.524 | 23 |
| B.1.639 | 23 |
| BA.5.2.37 | 23 |
| CK.2 | 23 |

| | |
|---|---|
| CV.1 | 23 |
| XAA | 23 |
| AY.93 | 22 |
| B.1.1.107 | 22 |
| B.1.1.130 | 22 |
| B.1.1.397 | 22 |
| B.1.1.480 | 22 |
| B.1.118 | 22 |
| B.1.12 | 22 |
| B.1.36.10 | 22 |
| BA.2.75.3 | 22 |
| BA.5.2.4 | 22 |
| N.3 | 22 |
| XAM | 22 |
| B.1.1.355 | 21 |
| B.1.1.359 | 21 |
| B.1.1.521 | 21 |
| B.1.258.10 | 21 |
| B.1.383 | 21 |
| B.1.395 | 21 |
| BA.2.28 | 21 |
| BA.2.58 | 21 |
| BF.23 | 21 |
| C.23 | 21 |
| C.36.3.1 | 21 |
| L.3 | 21 |
| XR | 21 |
| XY | 21 |
| AY.127.1 | 20 |
| AY.36.1 | 20 |
| B.1.1.122 | 20 |
| B.1.1.163 | 20 |
| B.1.1.323 | 20 |
| B.1.452 | 20 |
| B.1.544 | 20 |
| B.1.567 | 20 |
| B.1.636 | 20 |
| BE.4.1 | 20 |
| BE.4.2 | 20 |
| BL.2 | 20 |
| BR.1.2 | 20 |
| DF.1 | 20 |
| AT.1 | 19 |
| AY.123 | 19 |
| AY.21 | 19 |
| AY.43.6 | 19 |
| B.1.1.166 | 19 |
| B.1.1.218 | 19 |
| B.1.1.322 | 19 |
| B.1.1.363 | 19 |
| B.1.1.378 | 19 |
| B.1.131 | 19 |
| B.1.173 | 19 |
| B.1.177.38 | 19 |

| | |
|---|---|
| B.1.177.75 | 19 |
| B.1.206 | 19 |
| B.1.215 | 19 |
| B.1.306 | 19 |
| B.1.35 | 19 |
| B.1.428.3 | 19 |
| B.1.77 | 19 |
| B.13 | 19 |
| BA.4.5 | 19 |
| BQ.1.18 | 19 |
| BQ.1.4 | 19 |
| CR.1 | 19 |
| DF.1.1 | 19 |
| AY.43.7 | 18 |
| B.1.1.203 | 18 |
| B.1.1.328 | 18 |
| B.1.1.75 | 18 |
| B.1.1.97 | 18 |
| B.1.151 | 18 |
| B.1.177.23 | 18 |
| B.1.177.74 | 18 |
| B.1.310 | 18 |
| B.1.36.38 | 18 |
| B.1.592 | 18 |
| B.1.617.3 | 18 |
| BA.1.17.1 | 18 |
| BA.2.43 | 18 |
| BA.2.54 | 18 |
| BN.6 | 18 |
| CR.1.1 | 18 |
| EE.2 | 18 |
| B.1.1.120 | 17 |
| B.1.1.15 | 17 |
| B.1.1.412 | 17 |
| B.1.1.467 | 17 |
| B.1.1.86 | 17 |
| B.1.1.88 | 17 |
| B.1.115 | 17 |
| B.1.552 | 17 |
| B.1.570 | 17 |
| B.1.587 | 17 |
| B.18 | 17 |
| BA.2.3.7 | 17 |
| BA.5.1.16 | 17 |
| BA.5.2.47 | 17 |
| BF.12 | 17 |
| BF.38 | 17 |
| BN.1.2.1 | 17 |
| BQ.1.1.67 | 17 |
| CH.1.1.2 | 17 |
| CM.8.1 | 17 |
| U.1 | 17 |
| XBD | 17 |
| AY.78 | 16 |

| | |
|---|---|
| B.1.1.112 | 16 |
| B.1.1.133 | 16 |
| B.1.1.141 | 16 |
| B.1.1.308 | 16 |
| B.1.1.354 | 16 |
| B.1.1.371 | 16 |
| B.1.1.395 | 16 |
| B.1.1.46 | 16 |
| B.1.1.461 | 16 |
| B.1.231 | 16 |
| B.1.421 | 16 |
| B.1.521 | 16 |
| B.1.585 | 16 |
| B.1.604 | 16 |
| B.1.70 | 16 |
| B.51 | 16 |
| BA.2.3.11 | 16 |
| BA.2.3.16 | 16 |
| BA.2.38.1 | 16 |
| BC.2 | 16 |
| BQ.1.22 | 16 |
| CQ.1.1 | 16 |
| DK.1 | 16 |
| P.1.7.1 | 16 |
| A.26 | 15 |
| A.27 | 15 |
| AY.23.2 | 15 |
| AY.39.1.3 | 15 |
| AY.42.1 | 15 |
| AY.59 | 15 |
| AY.61 | 15 |
| B.1.1.285 | 15 |
| B.1.1.417 | 15 |
| B.1.1.57 | 15 |
| B.1.1.92 | 15 |
| B.1.128 | 15 |
| B.1.195 | 15 |
| B.1.199 | 15 |
| B.1.214.3 | 15 |
| B.1.218 | 15 |
| B.1.425 | 15 |
| B.1.637.1 | 15 |
| B.1.9.5 | 15 |
| B.4.7 | 15 |
| BA.5.2.36 | 15 |
| BA.5.2.56 | 15 |
| BM.1 | 15 |
| BU.2 | 15 |
| CL.1 | 15 |
| XAC | 15 |
| XAE | 15 |
| XBB.1.1 | 15 |
| A.29 | 14 |
| AJ.1 | 14 |

| | |
|---|---|
| AY.80 | 14 |
| B.1.1.109 | 14 |
| B.1.1.136 | 14 |
| B.1.1.428 | 14 |
| B.1.1.487 | 14 |
| B.1.1.528 | 14 |
| B.1.142 | 14 |
| B.1.189 | 14 |
| B.1.258.21 | 14 |
| B.1.277 | 14 |
| B.1.351.2 | 14 |
| B.1.377 | 14 |
| B.1.382 | 14 |
| B.1.415 | 14 |
| B.1.580 | 14 |
| B.1.81 | 14 |
| BA.1.14.2 | 14 |
| BA.5.1.8 | 14 |
| BA.5.2.62 | 14 |
| BF.7.16 | 14 |
| BF.7.20 | 14 |
| CK.3 | 14 |
| CN.1 | 14 |
| EE.5 | 14 |
| P.1.2 | 14 |
| XBB.3 | 14 |
| AY.29 | 13 |
| AY.91.1 | 13 |
| AY.99.1 | 13 |
| B.1.1.119 | 13 |
| B.1.1.159 | 13 |
| B.1.1.178 | 13 |
| B.1.1.266 | 13 |
| B.1.1.349 | 13 |
| B.1.1.367 | 13 |
| B.1.160.10 | 13 |
| B.1.177.21 | 13 |
| B.1.177.3 | 13 |
| B.1.177.59 | 13 |
| B.1.258.6 | 13 |
| B.1.407 | 13 |
| B.1.436 | 13 |
| B.1.543 | 13 |
| B.1.559 | 13 |
| B.1.603 | 13 |
| BA.1.16.2 | 13 |
| BA.1.3 | 13 |
| BA.2.38.2 | 13 |
| BA.2.79 | 13 |
| BA.4.6.2 | 13 |
| BA.4.8 | 13 |
| BA.5.1.28 | 13 |
| BA.5.2.14 | 13 |
| BF.29 | 13 |

| | |
|---|---|
| BF.7.23 | 13 |
| BQ.1.25.1 | 13 |
| BQ.2 | 13 |
| BV.2 | 13 |
| BZ.2 | 13 |
| CM.4 | 13 |
| CP.5 | 13 |
| DN.1 | 13 |
| L.4 | 13 |
| P.1.11 | 13 |
| AY.133 | 12 |
| B.1.1.139 | 12 |
| B.1.1.205 | 12 |
| B.1.1.243 | 12 |
| B.1.1.361 | 12 |
| B.1.1.421 | 12 |
| B.1.149 | 12 |
| B.1.160.16 | 12 |
| B.1.212 | 12 |
| B.1.213 | 12 |
| B.1.258.20 | 12 |
| B.1.315 | 12 |
| B.1.429.1 | 12 |
| B.1.431 | 12 |
| B.1.441 | 12 |
| B.1.549 | 12 |
| BA.2.30 | 12 |
| BA.4.6.3 | 12 |
| BA.5.1.34 | 12 |
| BN.5 | 12 |
| BQ.1.1.28 | 12 |
| BQ.1.1.43 | 12 |
| BR.2.1 | 12 |
| W.1 | 12 |
| XS | 12 |
| AY.104 | 11 |
| B.1.1.113 | 11 |
| B.1.1.254 | 11 |
| B.1.1.403 | 11 |
| B.1.1.47 | 11 |
| B.1.160.9 | 11 |
| B.1.177.35 | 11 |
| B.1.249 | 11 |
| B.1.294 | 11 |
| B.1.343 | 11 |
| B.1.359 | 11 |
| BA.2.3.1 | 11 |
| BA.2.75.4 | 11 |
| BA.4.3 | 11 |
| BF.11.2 | 11 |
| BF.20 | 11 |
| BF.24 | 11 |
| BF.7.22 | 11 |
| BQ.1.1.38 | 11 |

| | |
|---|---|
| C.36.1 | 11 |
| CD.2 | 11 |
| DV.3 | 11 |
| V.1 | 11 |
| A.16 | 10 |
| AY.25.1.2 | 10 |
| AY.4.14 | 10 |
| B.1.1.239 | 10 |
| B.1.1.302 | 10 |
| B.1.1.376 | 10 |
| B.1.1.38 | 10 |
| B.1.1.418 | 10 |
| B.1.1.48 | 10 |
| B.1.108 | 10 |
| B.1.160.20 | 10 |
| B.1.163 | 10 |
| B.1.177.63 | 10 |
| B.1.240.2 | 10 |
| B.1.245 | 10 |
| B.1.36.2 | 10 |
| B.1.448 | 10 |
| B.1.473 | 10 |
| B.1.474 | 10 |
| B.1.562 | 10 |
| B.1.563 | 10 |
| B.1.578 | 10 |
| B.1.640.1 | 10 |
| B.44 | 10 |
| B.47 | 10 |
| BA.2.60 | 10 |
| BA.4.1.5 | 10 |
| BQ.1.1.40 | 10 |
| BQ.1.1.66 | 10 |
| BQ.1.31 | 10 |
| CA.7 | 10 |
| CE.1 | 10 |
| CR.2 | 10 |
| EC.1 | 10 |
| XAG | 10 |
| XBB.1.9 | 10 |
| AY.28 | 9 |
| AY.34.1.1 | 9 |
| B.1.1.101 | 9 |
| B.1.1.118 | 9 |
| B.1.1.13 | 9 |
| B.1.1.155 | 9 |
| B.1.1.165 | 9 |
| B.1.1.298 | 9 |
| B.1.1.310 | 9 |
| B.1.1.400 | 9 |
| B.1.1.419 | 9 |
| B.1.1.56 | 9 |
| B.1.160.29 | 9 |
| B.1.177.66 | 9 |

| | |
|---|---|
| B.1.263 | 9 |
| B.1.267 | 9 |
| B.1.340 | 9 |
| B.1.36.34 | 9 |
| B.1.371 | 9 |
| B.1.390 | 9 |
| B.1.483 | 9 |
| B.1.510 | 9 |
| B.1.539 | 9 |
| B.1.541 | 9 |
| B.1.564 | 9 |
| B.1.564.1 | 9 |
| B.1.591 | 9 |
| BA.5.1.14 | 9 |
| BA.5.10.1 | 9 |
| BE.1.4.4 | 9 |
| BF.19 | 9 |
| BH.1 | 9 |
| BM.1.1.5 | 9 |
| BN.1.4.1 | 9 |
| BQ.1.1.16 | 9 |
| BQ.1.1.36 | 9 |
| BQ.1.1.62 | 9 |
| BQ.1.21 | 9 |
| BQ.1.7 | 9 |
| C.31 | 9 |
| P.3 | 9 |
| AA.1 | 8 |
| AY.122.5 | 8 |
| AY.98.1.1 | 8 |
| B.1.1.129 | 8 |
| B.1.1.132 | 8 |
| B.1.1.145 | 8 |
| B.1.1.147 | 8 |
| B.1.1.185 | 8 |
| B.1.1.226 | 8 |
| B.1.1.315 | 8 |
| B.1.1.344 | 8 |
| B.1.1.389 | 8 |
| B.1.1.413 | 8 |
| B.1.1.512 | 8 |
| B.1.1.58 | 8 |
| B.1.160.11 | 8 |
| B.1.160.14 | 8 |
| B.1.160.15 | 8 |
| B.1.243.2 | 8 |
| B.1.248 | 8 |
| B.1.256 | 8 |
| B.1.321 | 8 |
| B.1.330 | 8 |
| B.1.442 | 8 |
| B.1.491 | 8 |
| B.1.492 | 8 |
| B.1.542 | 8 |

| | |
|---|---|
| B.1.595.2 | 8 |
| B.36 | 8 |
| BA.2.12.2 | 8 |
| BA.2.77 | 8 |
| BA.5.1.20 | 8 |
| BA.5.2.12 | 8 |
| BA.5.2.58 | 8 |
| BF.3.1 | 8 |
| BF.7.24 | 8 |
| BF.7.26 | 8 |
| BF.7.8 | 8 |
| BQ.1.1.11 | 8 |
| BQ.1.1.13 | 8 |
| BQ.1.1.34 | 8 |
| BQ.1.1.53 | 8 |
| BQ.1.1.57 | 8 |
| BT.2 | 8 |
| BW.1 | 8 |
| CB.1 | 8 |
| N.1 | 8 |
| U.3 | 8 |
| AG.1 | 7 |
| AM.3 | 7 |
| AQ.1 | 7 |
| AY.39.1.1 | 7 |
| B.1.1.158 | 7 |
| B.1.1.162 | 7 |
| B.1.1.258 | 7 |
| B.1.1.261 | 7 |
| B.1.1.339 | 7 |
| B.1.1.399 | 7 |
| B.1.1.84 | 7 |
| B.1.160.12 | 7 |
| B.1.252 | 7 |
| B.1.350.1 | 7 |
| B.1.466.2 | 7 |
| B.1.619 | 7 |
| BA.2.19 | 7 |
| BA.2.66 | 7 |
| BA.2.69 | 7 |
| BA.4.1.4 | 7 |
| BA.5.2.32 | 7 |
| BA.5.2.38 | 7 |
| BA.5.2.39 | 7 |
| BA.5.3.4 | 7 |
| BA.5.6.3 | 7 |
| BF.11.3 | 7 |
| BF.11.4 | 7 |
| BF.7.4.2 | 7 |
| BN.1.10 | 7 |
| BQ.1.1.25 | 7 |
| BQ.1.2.1 | 7 |
| BQ.1.29 | 7 |
| BU.3 | 7 |

| | |
|---|---|
| BW.1.1 | 7 |
| BY.1.2 | 7 |
| C.2.1 | 7 |
| C.28 | 7 |
| C.4 | 7 |
| CD.1 | 7 |
| CH.1.1.9 | 7 |
| CM.5.2 | 7 |
| D.4 | 7 |
| DB.1 | 7 |
| DE.2 | 7 |
| DU.1 | 7 |
| P.1.12.1 | 7 |
| P.7 | 7 |
| XAJ | 7 |
| XAP | 7 |
| A.2.5.3 | 6 |
| AY.131 | 6 |
| AY.39.1.4 | 6 |
| AZ.5 | 6 |
| B.1.1.148 | 6 |
| B.1.1.172 | 6 |
| B.1.1.214 | 6 |
| B.1.1.275 | 6 |
| B.1.1.352 | 6 |
| B.1.1.358 | 6 |
| B.1.1.386 | 6 |
| B.1.1.402 | 6 |
| B.1.1.451 | 6 |
| B.1.1.525 | 6 |
| B.1.177.36 | 6 |
| B.1.177.39 | 6 |
| B.1.188 | 6 |
| B.1.258.18 | 6 |
| B.1.302 | 6 |
| B.1.333 | 6 |
| B.1.344 | 6 |
| B.1.37 | 6 |
| B.1.423 | 6 |
| B.1.513 | 6 |
| B.1.6 | 6 |
| B.1.88.1 | 6 |
| B.4.4 | 6 |
| B.6.5 | 6 |
| BA.1.2 | 6 |
| BA.2.70 | 6 |
| BA.2.75.7 | 6 |
| BA.5.1.26 | 6 |
| BA.5.2.10 | 6 |
| BA.5.2.30 | 6 |
| BA.5.6.4 | 6 |
| BE.6 | 6 |
| BE.8 | 6 |
| BE.9 | 6 |

| | |
|---|---|
| BF.7.11 | 6 |
| BG.1 | 6 |
| BG.4 | 6 |
| BN.1.9 | 6 |
| BN.2.1 | 6 |
| BQ.1.1.35 | 6 |
| BQ.1.1.42 | 6 |
| BQ.1.1.56 | 6 |
| BQ.1.28 | 6 |
| BQ.1.9 | 6 |
| BT.1 | 6 |
| CG.1 | 6 |
| CP.6 | 6 |
| CQ.1 | 6 |
| D.3 | 6 |
| DL.1 | 6 |
| G.1 | 6 |
| Q.7 | 6 |
| W.2 | 6 |
| XAN | 6 |
| XAU | 6 |
| XAY.1 | 6 |
| XBB.1.13 | 6 |
| XBF | 6 |
| AM.1 | 5 |
| AN.1 | 5 |
| AY.103.1 | 5 |
| AY.20.1 | 5 |
| AY.25.2 | 5 |
| AY.26.1 | 5 |
| AY.33.2 | 5 |
| AY.43.1 | 5 |
| AY.76 | 5 |
| B.1.1.135 | 5 |
| B.1.1.169 | 5 |
| B.1.1.187 | 5 |
| B.1.1.210 | 5 |
| B.1.1.34 | 5 |
| B.1.1.375 | 5 |
| B.1.1.398 | 5 |
| B.1.1.404 | 5 |
| B.1.1.450 | 5 |
| B.1.1.462 | 5 |
| B.1.1.500 | 5 |
| B.1.1.62 | 5 |
| B.1.170 | 5 |
| B.1.177.28 | 5 |
| B.1.243.1 | 5 |
| B.1.293 | 5 |
| B.1.348 | 5 |
| B.1.360 | 5 |
| B.1.381 | 5 |
| B.1.400.1 | 5 |
| B.1.438 | 5 |

| | |
|---|---|
| B.1.470 | 5 |
| B.1.478 | 5 |
| B.1.555 | 5 |
| B.1.569 | 5 |
| B.1.572 | 5 |
| B.1.601 | 5 |
| B.1.635 | 5 |
| B.5 | 5 |
| BA.1.13.1 | 5 |
| BA.2.61 | 5 |
| BA.2.75.6 | 5 |
| BA.2.9.6 | 5 |
| BF.11.5 | 5 |
| BF.18 | 5 |
| BF.7.12 | 5 |
| BF.7.19 | 5 |
| BQ.1.25 | 5 |
| BQ.1.27 | 5 |
| C.2 | 5 |
| CJ.1 | 5 |
| CM.9 | 5 |
| CP.1.1 | 5 |
| CT.1 | 5 |
| CZ.2 | 5 |
| DP.1 | 5 |
| EF.1.2 | 5 |
| N.9 | 5 |
| P.4 | 5 |
| XBB.1.7 | 5 |
| XBB.6 | 5 |
| A.25 | 4 |
| AK.2 | 4 |
| AY.75.2 | 4 |
| B.1.1.160 | 4 |
| B.1.1.29 | 4 |
| B.1.1.325 | 4 |
| B.1.1.341 | 4 |
| B.1.1.380 | 4 |
| B.1.1.381 | 4 |
| B.1.1.383 | 4 |
| B.1.1.40 | 4 |
| B.1.1.401 | 4 |
| B.1.1.524 | 4 |
| B.1.160.28 | 4 |
| B.1.167 | 4 |
| B.1.177.64 | 4 |
| B.1.178 | 4 |
| B.1.203 | 4 |
| B.1.208 | 4 |
| B.1.354 | 4 |
| B.1.450 | 4 |
| B.1.498 | 4 |
| B.1.547 | 4 |
| B.1.557 | 4 |

| | |
|---|---|
| B.1.621.2 | 4 |
| B.1.69 | 4 |
| B.32 | 4 |
| B.4.8 | 4 |
| B.49 | 4 |
| BA.2.10.4 | 4 |
| BA.2.38.3 | 4 |
| BA.5.2.41 | 4 |
| BA.5.2.63 | 4 |
| BE.4.1.1 | 4 |
| BF.30 | 4 |
| BF.7.10 | 4 |
| BF.7.18 | 4 |
| BG.3 | 4 |
| BL.1.4 | 4 |
| BL.3 | 4 |
| BN.3.1 | 4 |
| BQ.1.1.29 | 4 |
| BQ.1.1.47 | 4 |
| BQ.1.1.58 | 4 |
| BQ.1.1.63 | 4 |
| BV.1 | 4 |
| BW.1.1.1 | 4 |
| C.18 | 4 |
| CA.3 | 4 |
| CP.1.2 | 4 |
| Q.6 | 4 |
| Q.8 | 4 |
| V.2 | 4 |
| XBB.1.4 | 4 |
| XBB.2.2 | 4 |
| XF | 4 |
| A.11 | 3 |
| A.6 | 3 |
| AA.7 | 3 |
| AY.16.1 | 3 |
| AY.23.1 | 3 |
| AY.4.15 | 3 |
| AY.46.3 | 3 |
| AY.82 | 3 |
| B.1.1.221 | 3 |
| B.1.1.244 | 3 |
| B.1.1.263 | 3 |
| B.1.1.280 | 3 |
| B.1.1.331 | 3 |
| B.1.1.347 | 3 |
| B.1.1.356 | 3 |
| B.1.1.429 | 3 |
| B.1.1.438 | 3 |
| B.1.1.459 | 3 |
| B.1.1.99 | 3 |
| B.1.110.2 | 3 |
| B.1.146 | 3 |
| B.1.159 | 3 |

| | |
|---|---|
| B.1.160.31 | 3 |
| B.1.177.24 | 3 |
| B.1.177.34 | 3 |
| B.1.198 | 3 |
| B.1.258.14 | 3 |
| B.1.323 | 3 |
| B.1.355 | 3 |
| B.1.36.26 | 3 |
| B.1.362.1 | 3 |
| B.1.363 | 3 |
| B.1.372 | 3 |
| B.1.44 | 3 |
| B.1.466 | 3 |
| B.1.479 | 3 |
| B.1.501 | 3 |
| B.1.504 | 3 |
| B.1.550 | 3 |
| B.1.571 | 3 |
| B.1.629 | 3 |
| B.1.640 | 3 |
| B.38 | 3 |
| BA.2.75.10 | 3 |
| BA.4.1.10 | 3 |
| BA.4.1.3 | 3 |
| BA.5.1.31 | 3 |
| BA.5.1.37 | 3 |
| BA.5.2.52 | 3 |
| BA.5.2.55 | 3 |
| BE.1.4.3 | 3 |
| BE.10 | 3 |
| BF.17 | 3 |
| BF.7.1 | 3 |
| BF.7.3 | 3 |
| BM.2 | 3 |
| BN.1.3.3 | 3 |
| BN.1.3.5 | 3 |
| BN.4 | 3 |
| BQ.1.1.17 | 3 |
| BQ.1.1.54 | 3 |
| BQ.1.24 | 3 |
| BQ.1.26.1 | 3 |
| C.20 | 3 |
| C.26 | 3 |
| C.36 | 3 |
| C.36.2 | 3 |
| C.9 | 3 |
| CK.1.2 | 3 |
| CM.12 | 3 |
| CM.4.1 | 3 |
| CM.5 | 3 |
| DA.1 | 3 |
| ED.3 | 3 |
| M.3 | 3 |
| P.1.4 | 3 |

| | |
|---|---|
| P.1.9 | 3 |
| XAB | 3 |
| XAF | 3 |
| XBB.1.5.2 | 3 |
| XBB.3.1 | 3 |
| XBC.1 | 3 |
| XBG | 3 |
| XBM | 3 |
| XBN | 3 |
| XU | 3 |
| A.18 | 2 |
| A.23 | 2 |
| AA.4 | 2 |
| AE.1 | 2 |
| AY.125.1 | 2 |
| AY.31 | 2 |
| AY.4.12 | 2 |
| AY.4.17 | 2 |
| AY.46.6.1 | 2 |
| AY.5.6 | 2 |
| AY.75.3 | 2 |
| AY.9.2.2 | 2 |
| B.1.1.182 | 2 |
| B.1.1.27 | 2 |
| B.1.1.288 | 2 |
| B.1.1.350 | 2 |
| B.1.1.353 | 2 |
| B.1.1.425 | 2 |
| B.1.1.437 | 2 |
| B.1.1.440 | 2 |
| B.1.1.453 | 2 |
| B.1.1.458 | 2 |
| B.1.106 | 2 |
| B.1.110.1 | 2 |
| B.1.116 | 2 |
| B.1.160.19 | 2 |
| B.1.177.12 | 2 |
| B.1.233 | 2 |
| B.1.240.1 | 2 |
| B.1.258.9 | 2 |
| B.1.279 | 2 |
| B.1.334 | 2 |
| B.1.341 | 2 |
| B.1.358 | 2 |
| B.1.385 | 2 |
| B.1.406 | 2 |
| B.1.411 | 2 |
| B.1.451 | 2 |
| B.1.466.1 | 2 |
| B.1.475 | 2 |
| B.1.489 | 2 |
| B.1.494 | 2 |
| B.1.507 | 2 |
| B.1.508 | 2 |

| | |
|---|---|
| B.1.518 | 2 |
| B.1.520 | 2 |
| B.1.586 | 2 |
| B.1.590 | 2 |
| B.1.595.1 | 2 |
| B.1.619.1 | 2 |
| B.1.640.2 | 2 |
| B.1.641 | 2 |
| B.1.9.4 | 2 |
| B.1.94 | 2 |
| B.42 | 2 |
| B.60 | 2 |
| BA.2.3.8 | 2 |
| BA.2.38.4 | 2 |
| BA.2.79.1 | 2 |
| BA.2.82 | 2 |
| BA.2.9.4 | 2 |
| BA.5.7 | 2 |
| BC.1 | 2 |
| BF.31.1 | 2 |
| BF.41.1 | 2 |
| BF.7.5.1 | 2 |
| BJ.1 | 2 |
| BL.6 | 2 |
| BM.1.1.1 | 2 |
| BQ.1.1.26 | 2 |
| BQ.1.1.30 | 2 |
| BQ.1.1.44 | 2 |
| BQ.1.1.60 | 2 |
| BQ.1.1.61 | 2 |
| BQ.1.20 | 2 |
| BR.2 | 2 |
| BR.5 | 2 |
| CA.3.1 | 2 |
| CH.1.1.8 | 2 |
| CY.1 | 2 |
| DB.2 | 2 |
| DG.1 | 2 |
| DT.1 | 2 |
| P.1.17.1 | 2 |
| P.5 | 2 |
| XAH | 2 |
| XAL | 2 |
| XAY.3 | 2 |
| XBB.1.10 | 2 |
| XBB.1.11 | 2 |
| XBB.1.5.7 | 2 |
| XBB.3.3 | 2 |
| XH | 2 |
| A.15 | 1 |
| A.30 | 1 |
| AA.3 | 1 |
| AA.6 | 1 |
| AC.1 | 1 |

| | |
|---|---|
| AE.3 | 1 |
| AE.4 | 1 |
| AE.5 | 1 |
| AH.2 | 1 |
| AY.102.1 | 1 |
| AY.112.1 | 1 |
| AY.123.1 | 1 |
| AY.132 | 1 |
| AY.24.1 | 1 |
| AY.29.1 | 1 |
| AY.69 | 1 |
| B.1.1.114 | 1 |
| B.1.1.116 | 1 |
| B.1.1.127 | 1 |
| B.1.1.152 | 1 |
| B.1.1.177 | 1 |
| B.1.1.190 | 1 |
| B.1.1.193 | 1 |
| B.1.1.229 | 1 |
| B.1.1.242 | 1 |
| B.1.1.265 | 1 |
| B.1.1.268 | 1 |
| B.1.1.284 | 1 |
| B.1.1.335 | 1 |
| B.1.1.357 | 1 |
| B.1.1.370 | 1 |
| B.1.1.373 | 1 |
| B.1.1.379 | 1 |
| B.1.1.388 | 1 |
| B.1.1.405 | 1 |
| B.1.1.410 | 1 |
| B.1.1.426 | 1 |
| B.1.1.441 | 1 |
| B.1.1.447 | 1 |
| B.1.1.463 | 1 |
| B.1.1.465 | 1 |
| B.1.1.482 | 1 |
| B.1.1.514 | 1 |
| B.1.1.72 | 1 |
| B.1.1.77 | 1 |
| B.1.1.82 | 1 |
| B.1.119 | 1 |
| B.1.120 | 1 |
| B.1.134 | 1 |
| B.1.143 | 1 |
| B.1.160.13 | 1 |
| B.1.177.29 | 1 |
| B.1.177.31 | 1 |
| B.1.177.41 | 1 |
| B.1.177.42 | 1 |
| B.1.177.45 | 1 |
| B.1.177.61 | 1 |
| B.1.177.70 | 1 |
| B.1.177.71 | 1 |

| | |
|---|---|
| B.1.194 | 1 |
| B.1.205 | 1 |
| B.1.214 | 1 |
| B.1.219 | 1 |
| B.1.221.2 | 1 |
| B.1.224 | 1 |
| B.1.258.11 | 1 |
| B.1.291 | 1 |
| B.1.309 | 1 |
| B.1.318 | 1 |
| B.1.38 | 1 |
| B.1.387 | 1 |
| B.1.393 | 1 |
| B.1.417 | 1 |
| B.1.420 | 1 |
| B.1.424 | 1 |
| B.1.428.1 | 1 |
| B.1.428.2 | 1 |
| B.1.432 | 1 |
| B.1.438.3 | 1 |
| B.1.462 | 1 |
| B.1.467 | 1 |
| B.1.482 | 1 |
| B.1.487 | 1 |
| B.1.495 | 1 |
| B.1.497 | 1 |
| B.1.527 | 1 |
| B.1.528 | 1 |
| B.1.530 | 1 |
| B.1.531 | 1 |
| B.1.535 | 1 |
| B.1.545 | 1 |
| B.1.566 | 1 |
| B.1.574 | 1 |
| B.1.595.4 | 1 |
| B.1.606 | 1 |
| B.1.612 | 1 |
| B.1.615 | 1 |
| B.1.83 | 1 |
| B.1.9.2 | 1 |
| B.12 | 1 |
| B.30 | 1 |
| B.37 | 1 |
| B.41 | 1 |
| B.43 | 1 |
| B.6.3 | 1 |
| BA.1.24 | 1 |
| BA.2.85 | 1 |
| BA.5.1.32 | 1 |
| BA.5.2.46 | 1 |
| BA.5.2.60 | 1 |
| BF.10.1 | 1 |
| BF.34 | 1 |
| BF.38.1 | 1 |

| | | |
|---|---|---|
| BF.5.4 | 1 | |
| BF.7.13.2 | | 1 |
| BF.7.14 | 1 | |
| BF.7.15 | 1 | |
| BF.7.19.1 | | 1 |
| BF.7.9 | 1 | |
| BL.1.3 | 1 | |
| BM.2.1 | 1 | |
| BM.3 | 1 | |
| BM.4 | 1 | |
| BM.5 | 1 | |
| BM.6 | 1 | |
| BN.1.1 | 1 | |
| BN.1.1.1 | | 1 |
| BN.1.3.4 | | 1 |
| BQ.1.1.14 | | 1 |
| BQ.1.1.20 | | 1 |
| BQ.1.1.21 | | 1 |
| BQ.1.1.37 | | 1 |
| BQ.1.1.46 | | 1 |
| BQ.1.1.48 | | 1 |
| BQ.1.1.55 | | 1 |
| BQ.1.1.65 | | 1 |
| BQ.1.1.70 | | 1 |
| BQ.1.1.9 | | 1 |
| BQ.1.10.2 | | 1 |
| BQ.1.17 | 1 | |
| BQ.1.19 | 1 | |
| BR.3 | 1 | |
| BS.1 | 1 | |
| BS.1.1 | 1 | |
| BW.1.1.2 | | 1 |
| BY.1.1 | 1 | |
| BY.1.1.1 | | 1 |
| BZ.1 | 1 | |
| C.29 | 1 | |
| C.33 | 1 | |
| C.34 | 1 | |
| C.40 | 1 | |
| C.6 | 1 | |
| CA.2 | 1 | |
| CC.1 | 1 | |
| CL.1.3 | 1 | |
| CM.1 | 1 | |
| CM.3 | 1 | |
| CM.5.1 | 1 | |
| CM.7 | 1 | |
| CP.2 | 1 | |
| CP.4 | 1 | |
| CR.1.3 | 1 | |
| DJ.1.1.1 | | 1 |
| DQ.1 | 1 | |
| DR.1 | 1 | |
| EC.1.1 | 1 | |

| | |
|---|---|
| EE.3 | 1 |
| N.4 | 1 |
| P.1.5 | 1 |
| P.1.8 | 1 |
| Q.2 | 1 |
| R.2 | 1 |
| XAD | 1 |
| XAK | 1 |
| XAV | 1 |
| XAY | 1 |
| XBB.1.4.1 | 1 |
| XBB.1.5.1 | 1 |
| XBB.1.5.3 | 1 |
| XBB.1.5.4 | 1 |
| XBB.1.5.5 | 1 |
| XBB.1.5.6 | 1 |
| XBB.1.5.8 | 1 |
| XBB.2.1 | 1 |
| XBB.2.3 | 1 |
| XBB.4 | 1 |
| XBB.5 | 1 |
| XBB.6.1 | 1 |
| XBB.7 | 1 |
| XBC.1.1 | 1 |
| XBK | 1 |
| XG | 1 |

WHO Lineages for Submitted Sequences

| WHO Lineages | WHO Lineages counts |
| --- | --- |
| Delta | 2502648 |
| Omicron | 1747736 |
| Unassigned | 1074048 |
| Alpha | 542067 |
| Probable | 66206 |
| Epsilon | 40079 |
| A.23.1-like | 224 |
| A.23.1-like+E484K | 53 |
| AV.1-like | 165 |
| B.1.1.318-like | 2305 |
| B.1.1.7-like+E484K | 1086 |
| B.1.617.1-like | 843 |
| B.1.617.3-like | 14 |
| Beta | 7482 |
| Eta | 2456 |
| Gamma | 22763 |
| Lambda | 1298 |
| Mu | 5352 |
| Theta | 45 |
| Zeta | 1181 |
| Iota | 33030 |

WHO Lineages for the Systematic Analysis

| WHO Lineages | WHO Lineages counts |
| --- | --- |
| Delta | 1000395 |
| Omicron | 866575 |
| Unassigned | 171741 |
| Alpha | 138094 |
| Epsilon | 16151 |
| Iota | 14988 |
| A.23.1-like | 115 |
| A.23.1-like+E484K | 22 |
| AV.1-like | 28 |
| B.1.1.318-like | 1556 |
| B.1.1.7-like+E484K | 325 |
| B.1.617.1-like | 447 |
| B.1.617.3-like | 17 |
| Beta | 3487 |
| Eta | 682 |
| Gamma | 9813 |
| Lambda | 587 |
| Mu | 2880 |
| Theta | 9 |
| Zeta | 523 |
| Probable Omicron | 11292 |

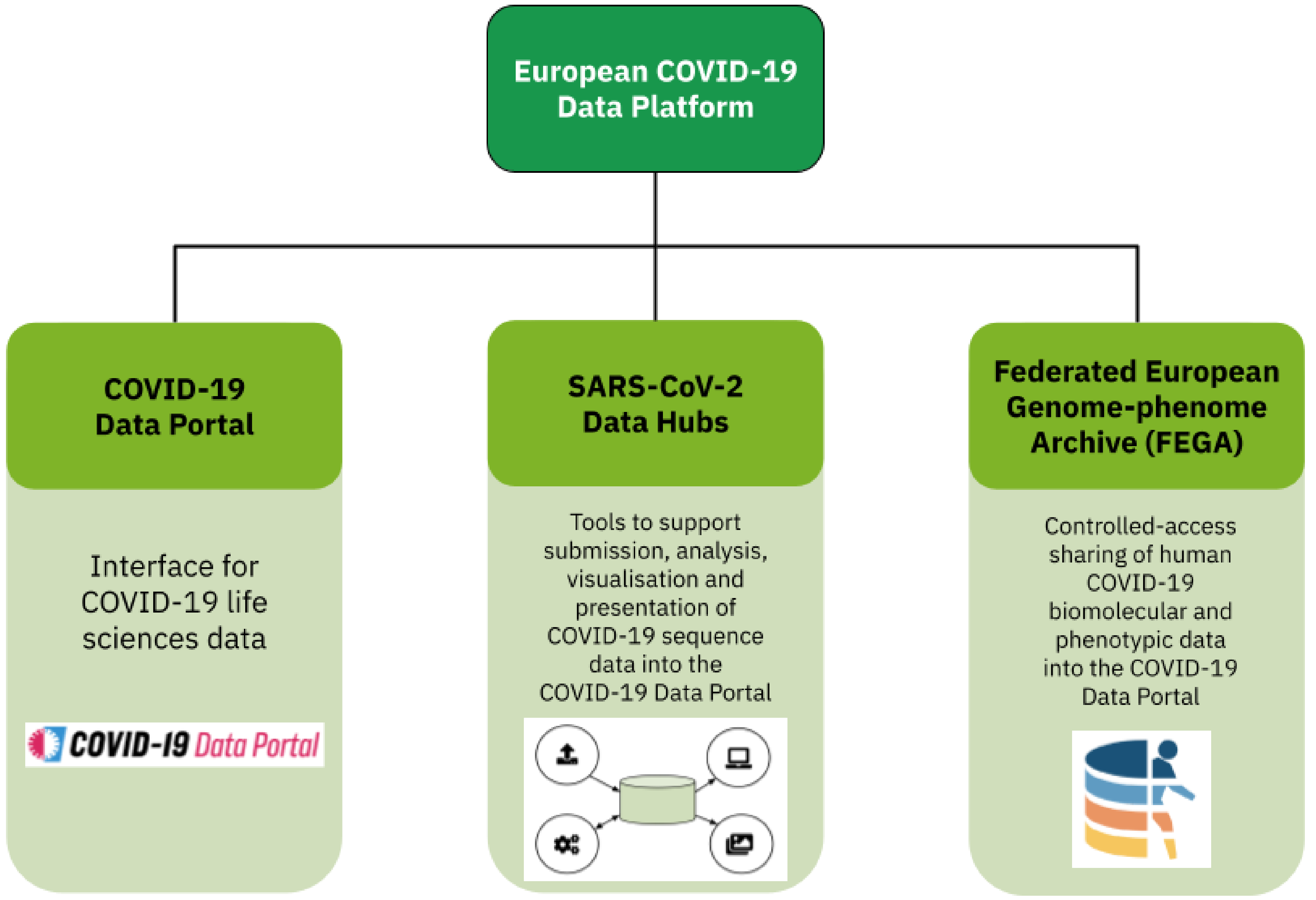

European COVID-19 Data Platform
COVID-19 Data Portal
Interface for COVID-19 life sciences data
COVID-19 Data Portal
SARS-CoV-2 Data Hubs
Tools to support submission, analysis, visualisation and presentation of COVID-19 sequence data into the COVID-19 Data Portal
Federated European Genome-phenome Archive (FEGA)
Controlled-access sharing of human COVID-19 biomolecular and phenotypic data into the COVID-19 Data Portal

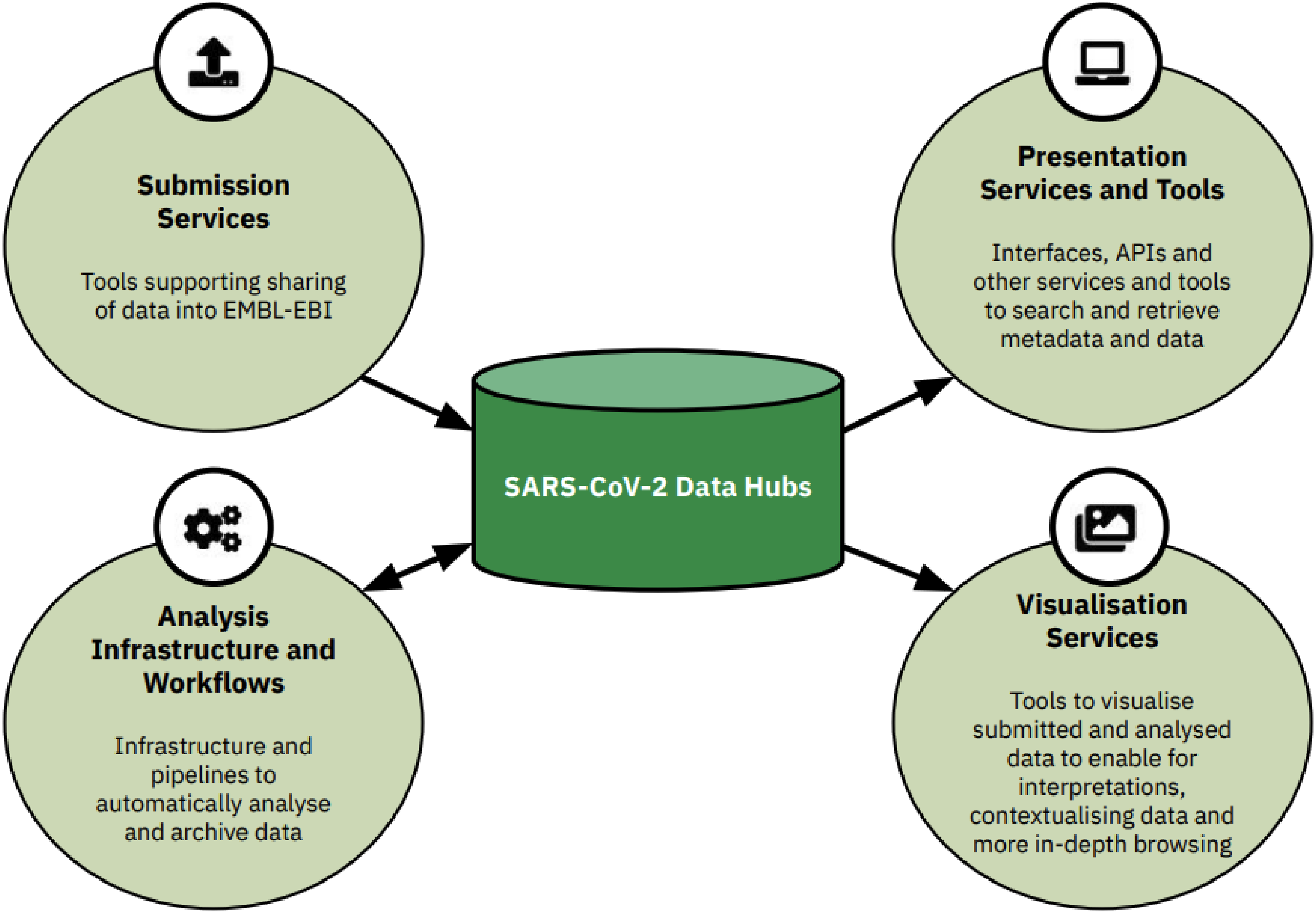
Submission Services
Tools supporting sharing of data into EMBL-EBI
Presentation Services and Tools
Interfaces, APIs and other services and tools to search and retrieve metadata and data
SARS-CoV-2 Data Hubs
Analysis Infrastructure and Workflows
Infrastructure and pipelines to automatically analyse and archive data
Visualisation Services
Tools to visualise submitted and analysed data to enable for interpretations, contextualising data and more in-depth browsing

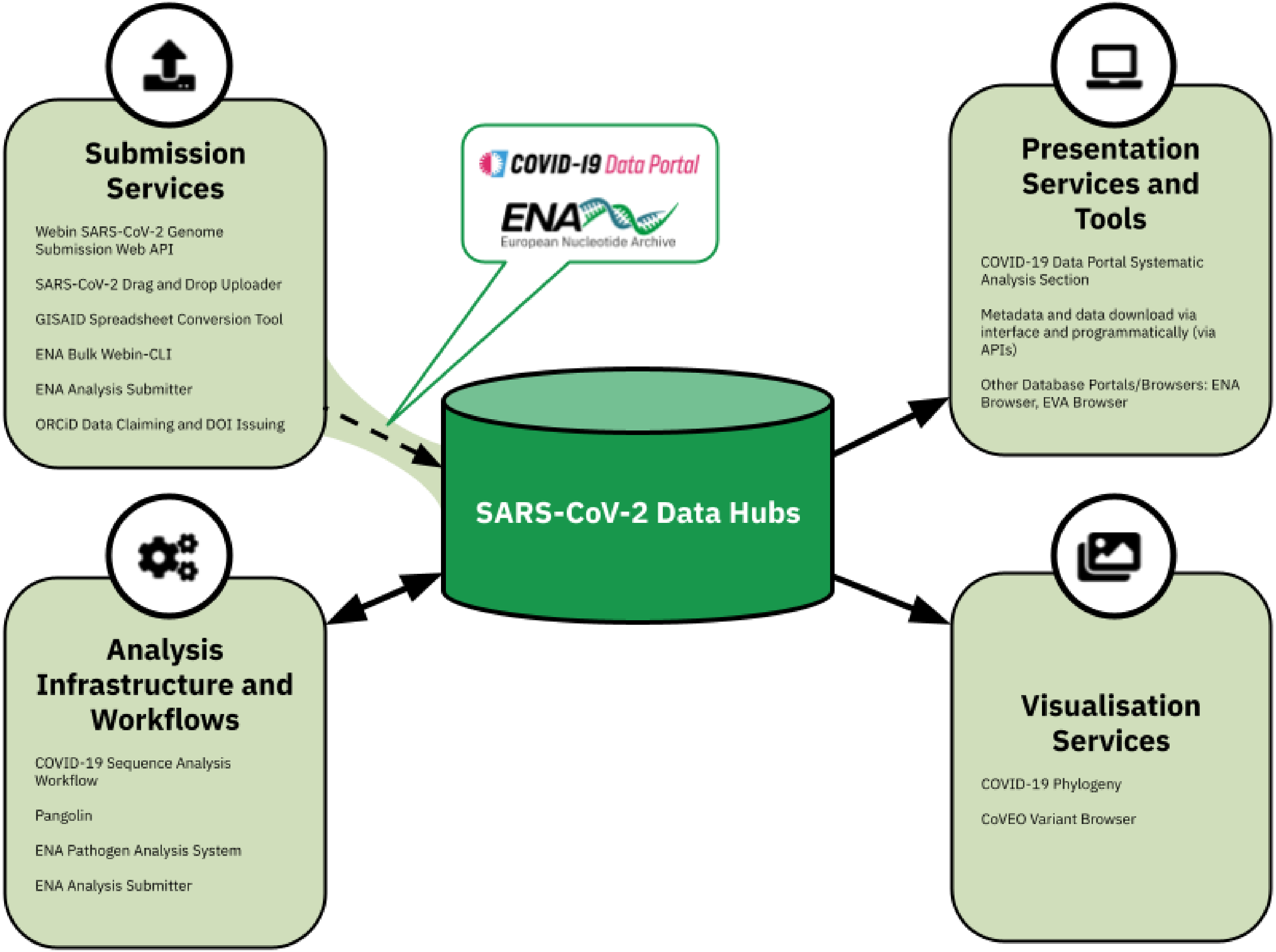

Submission Services
Webin SARS-CoV-2 Genome Submission Web API
SARS-CoV-2 Drag and Drop Uploader
GISAID Spreadsheet Conversion Tool
ENA Bulk Webin-CLI
ENA Analysis Submitter
ORCiD Data Claiming and DOI Issuing
COVID-19 Data Portal
ENA
European Nucleotide Archive
Presentation Services and Tools
COVID-19 Data Portal Systematic Analysis Section
Metadata and data download via interface and programmatically (via APIs)
Other Database Portals/Browsers: ENA Browser, EVA Browser
SARS-CoV-2 Data Hubs
Analysis Infrastructure and Workflows
COVID-19 Sequence Analysis Workflow
Pangolin
ENA Pathogen Analysis System
ENA Analysis Submitter
Visualisation Services
COVID-19 Phylogeny
CoVEO Variant Browser

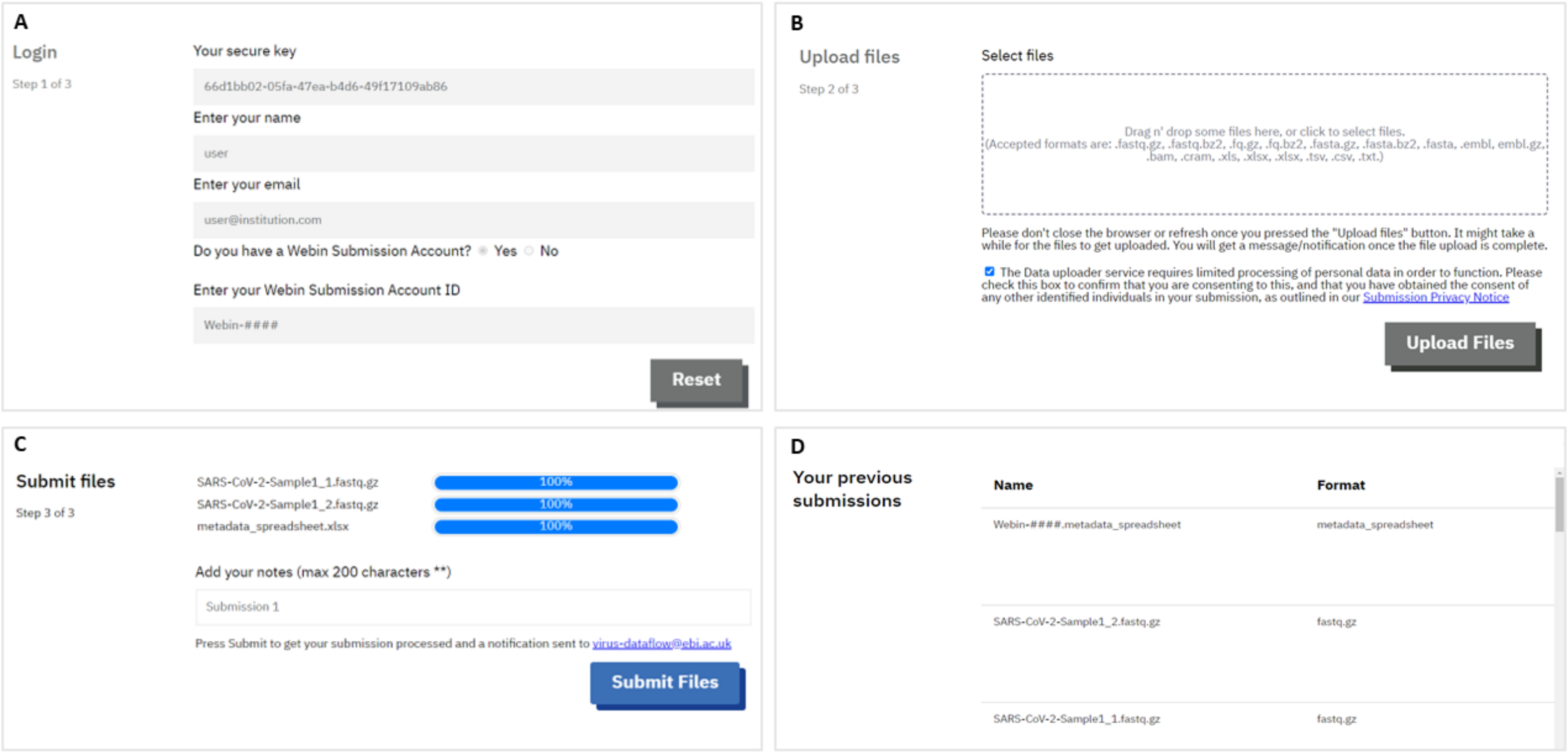
A
Login
Step 1 of 3
Your secure key
66d1bb02-05fa-47ea-b4d6-49f17109ab86
Enter your name
user
Enter your email
user@institution.com
Do you have a Webin Submission Account? Yes No
Enter your Webin Submission Account ID
Webin-####
Reset
B
Upload files
Step 2 of 3
Select files
Drag n' drop some files here, or click to select files.
(Accepted formats are: .fastq.gz, .fastq.bz2, .fq.gz, .fq.bz2, .fasta.gz, .fasta.bz2, .fasta, .embl, embl.gz, .bam, .cram, .xls, .xlsx, .xlsx, .tsv, .csv, .txt.)
Please don't close the browser or refresh once you pressed the "Upload files" button. It might take a while for the files to get uploaded. You will get a message/notification once the file upload is complete.
The Data uploader service requires limited processing of personal data in order to function. Please check this box to confirm that you are consenting to this, and that you have obtained the consent of any other identified individuals in your submission, as outlined in our Submission Privacy Notice
Upload Files
C
Submit files
Step 3 of 3
SARS-CoV-2-Sample1_1.fastq.gz 100%
SARS-CoV-2-Sample1_2.fastq.gz 100%
metadata_spreadsheet.xlsx 100%
Add your notes (max 200 characters **)
Submission 1
Press Submit to get your submission processed and a notification sent to virus-dataflow@ebi.ac.uk
Submit Files
D
Your previous submissions
Name
Format
Webin-####.metadata_spreadsheet
metadata_spreadsheet
SARS-CoV-2-Sample1_2.fastq.gz
fastq.gz
SARS-CoV-2-Sample1_1.fastq.gz
fastq.gz

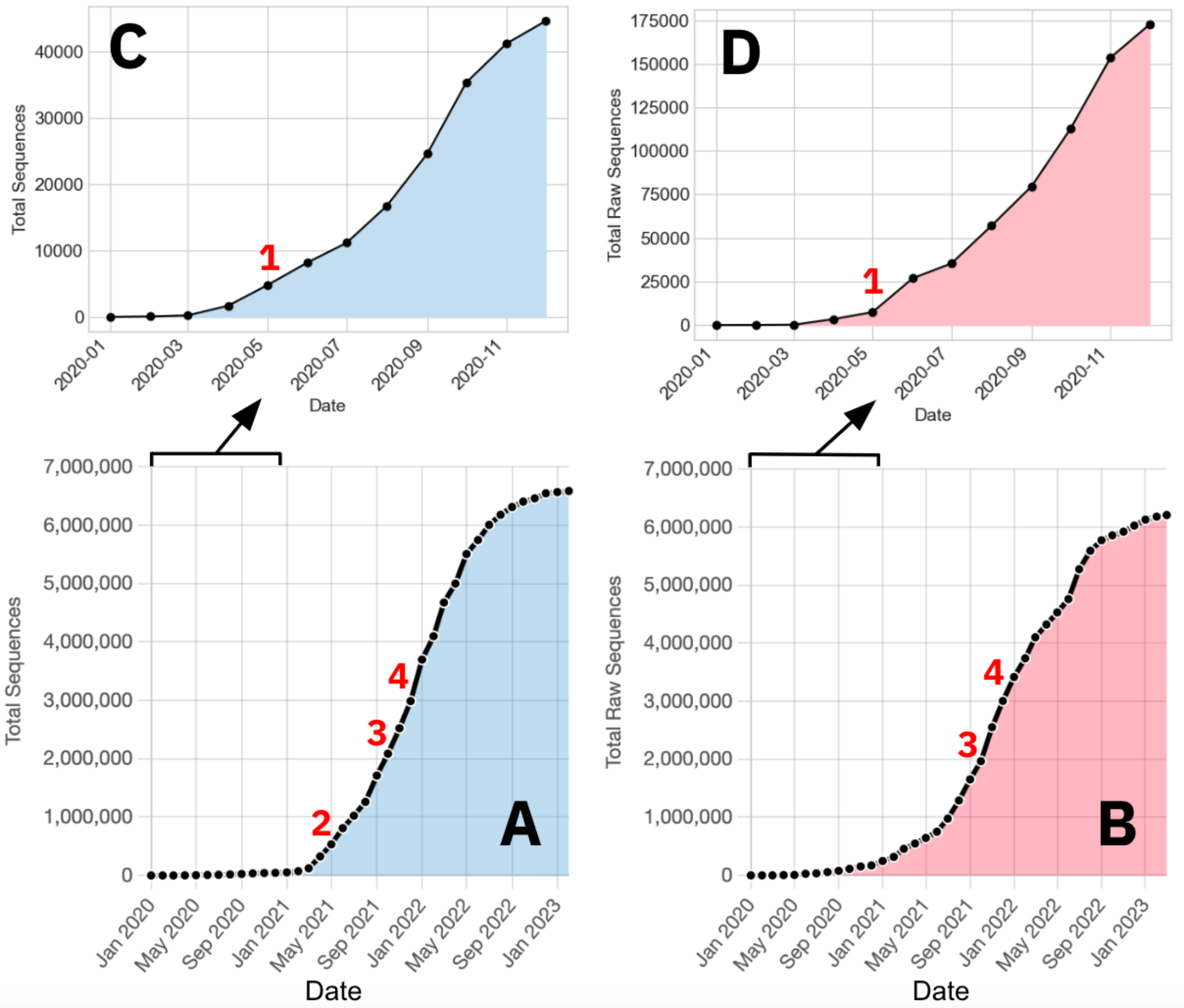
C
Total Sequences
0
10000
20000
30000
40000
2020-01
2020-03
2020-05
2020-07
2020-09
2020-11
Date
1
D
Total Raw Sequences
0
25000
50000
75000
100000
125000
150000
175000
2020-01
2020-03
2020-05
2020-07
2020-09
2020-11
Date
1
A
Total Sequences
0
1,000,000
2,000,000
3,000,000
4,000,000
5,000,000
6,000,000
7,000,000
Jan 2020
May 2020
Sep 2020
Jan 2021
May 2021
Sep 2021
Jan 2022
May 2022
Sep 2022
Jan 2023
Date
2
3
4
B
Total Raw Sequences
0
1,000,000
2,000,000
3,000,000
4,000,000
5,000,000
6,000,000
7,000,000
Jan 2020
May 2020
Sep 2020
Jan 2021
May 2021
Sep 2021
Jan 2022
May 2022
Sep 2022
Jan 2023
Date
3
4

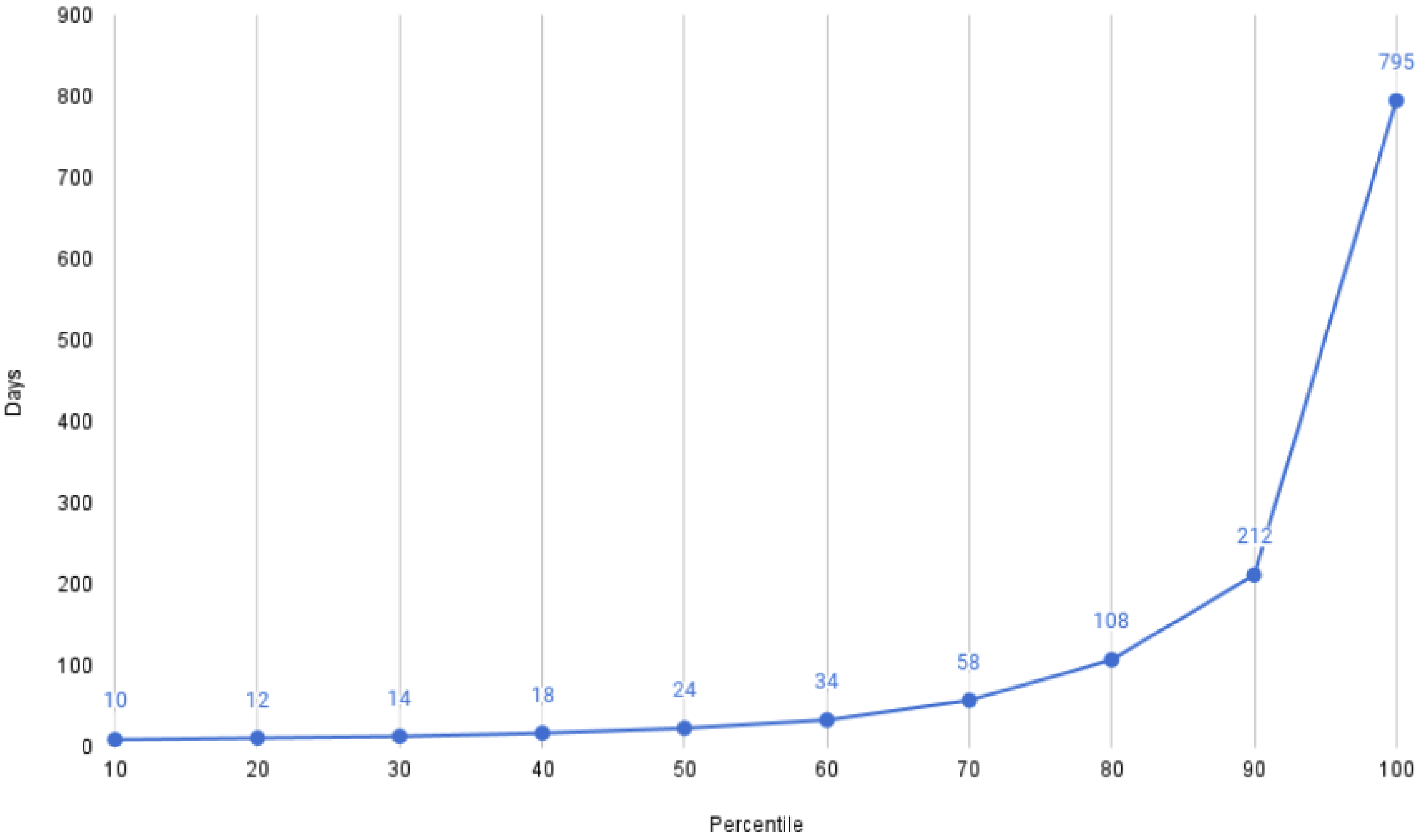

Days
Percentile
900
800
700
600
500
400
300
200
100
0
10
20
30
40
50
60
70
80
90
100
10
12
14
18
24
34
58
108
212
795

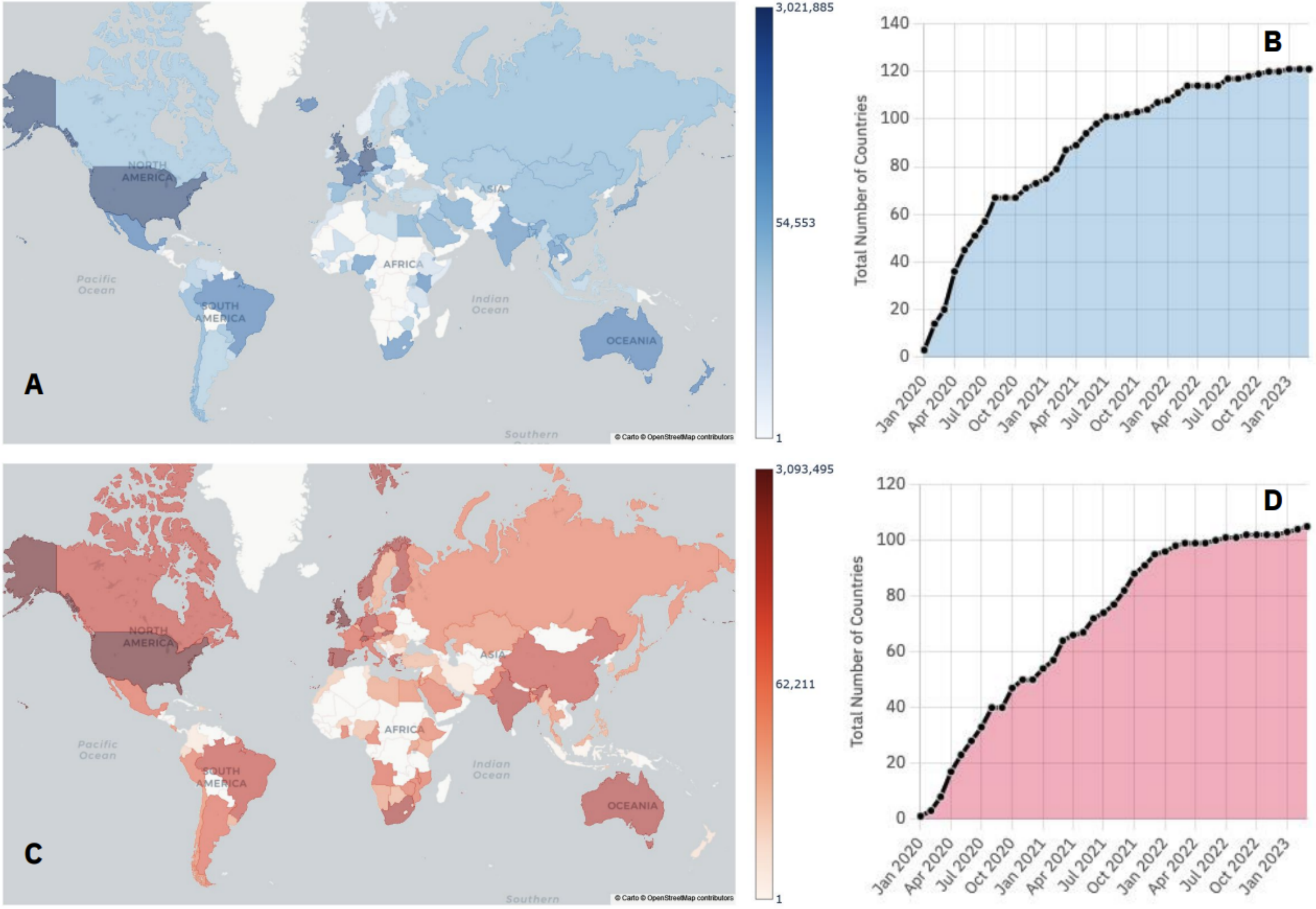
A
NORTH AMERICA
SOUTH AMERICA
ASIA
AFRICA
OCEANIA
Pacific Ocean
Indian Ocean
Southern
© Carto © OpenStreetMap contributors
3,021,885
54,553
1
B
Total Number of Countries
0
20
40
60
80
100
120
140
Jan 2020
Apr 2020
Jul 2020
Oct 2020
Jan 2021
Apr 2021
Jul 2021
Oct 2021
Jan 2022
Apr 2022
Jul 2022
Oct 2022
Jan 2023
C
NORTH AMERICA
SOUTH AMERICA
ASIA
AFRICA
OCEANIA
Pacific Ocean
Indian Ocean
Southern
© Carto © OpenStreetMap contributors
3,093,495
62,211
1
D
Total Number of Countries
0
20
40
60
80
100
120
Jan 2020
Apr 2020
Jul 2020
Oct 2020
Jan 2021
Apr 2021
Jul 2021
Oct 2021
Jan 2022
Apr 2022
Jul 2022
Oct 2022
Jan 2023

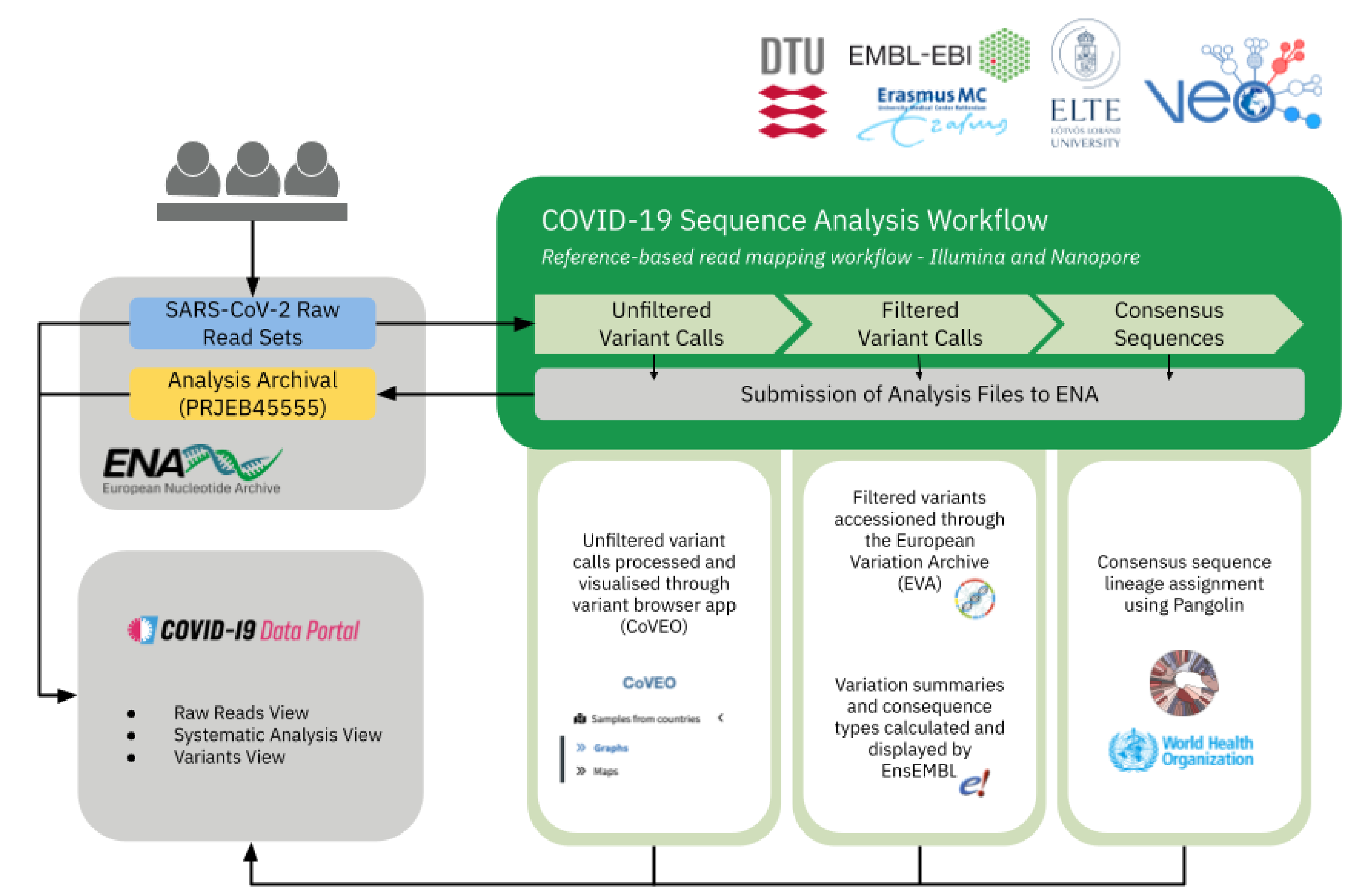
DTU
EMBL-EBI
Erasmus MC
ELTE
EÖTVÖS LORÁND UNIVERSITY
VEO
SARS-CoV-2 Raw Read Sets
Analysis Archival (PRJEB45555)
ENA
European Nucleotide Archive
COVID-19 Sequence Analysis Workflow
Reference-based read mapping workflow - Illumina and Nanopore
Unfiltered Variant Calls
Filtered Variant Calls
Consensus Sequences
Submission of Analysis Files to ENA
Unfiltered variant calls processed and visualised through variant browser app (CoVEO)
CoVEO
Samples from countries
Graphs
Maps
Filtered variants accessioned through the European Variation Archive (EVA)
Variation summaries and consequence types calculated and displayed by EnsEMBL
Consensus sequence lineage assignment using Pangolin
World Health Organization
COVID-19 Data Portal
Raw Reads View
Systematic Analysis View
Variants View

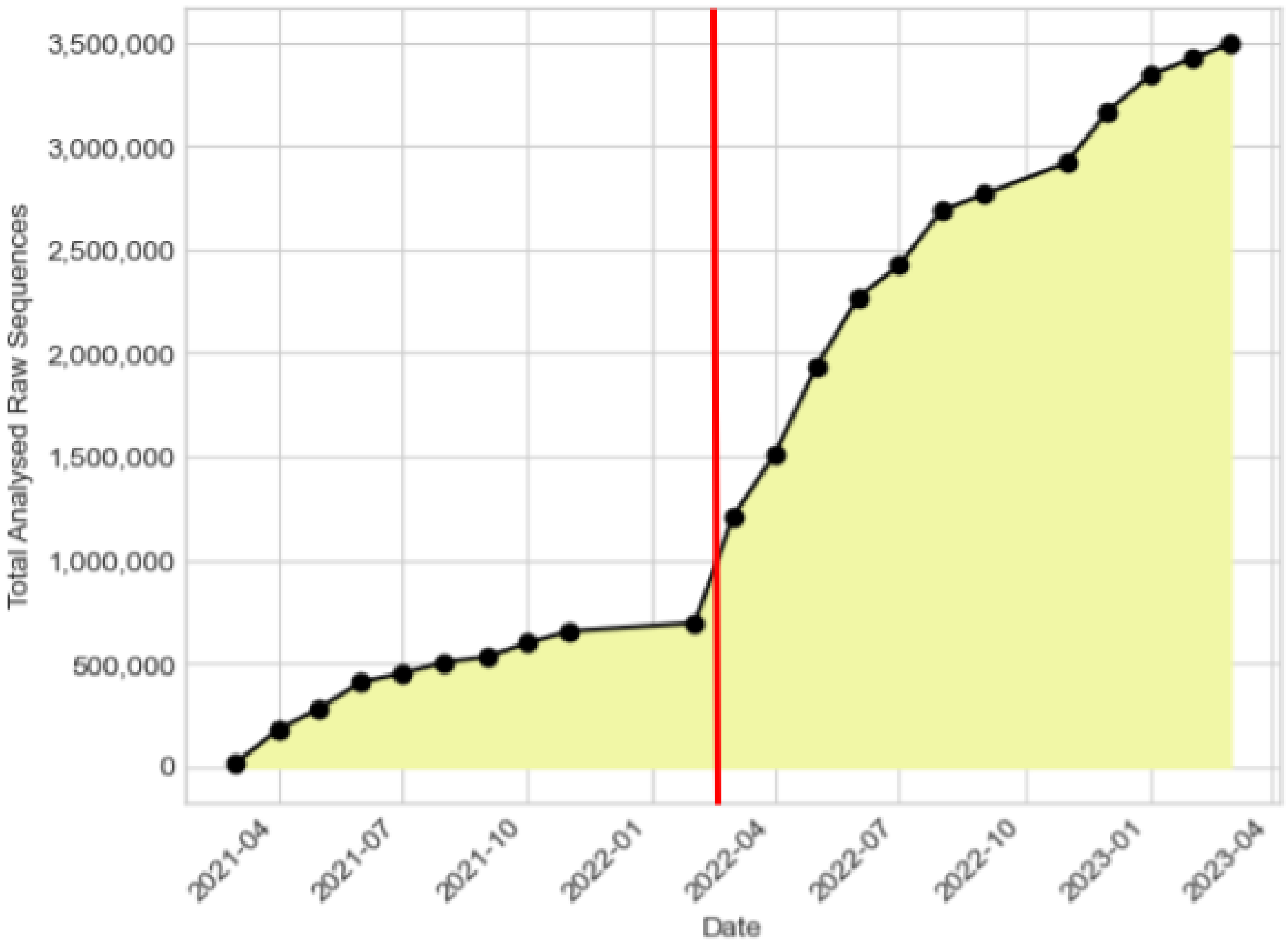

3,500,000
3,000,000
2,500,000
2,000,000
1,500,000
1,000,000
500,000
0
Total Analysed Raw Sequences
2021-04
2021-07
2021-10
2022-01
2022-04
2022-07
2022-10
2023-01
2023-04
Date

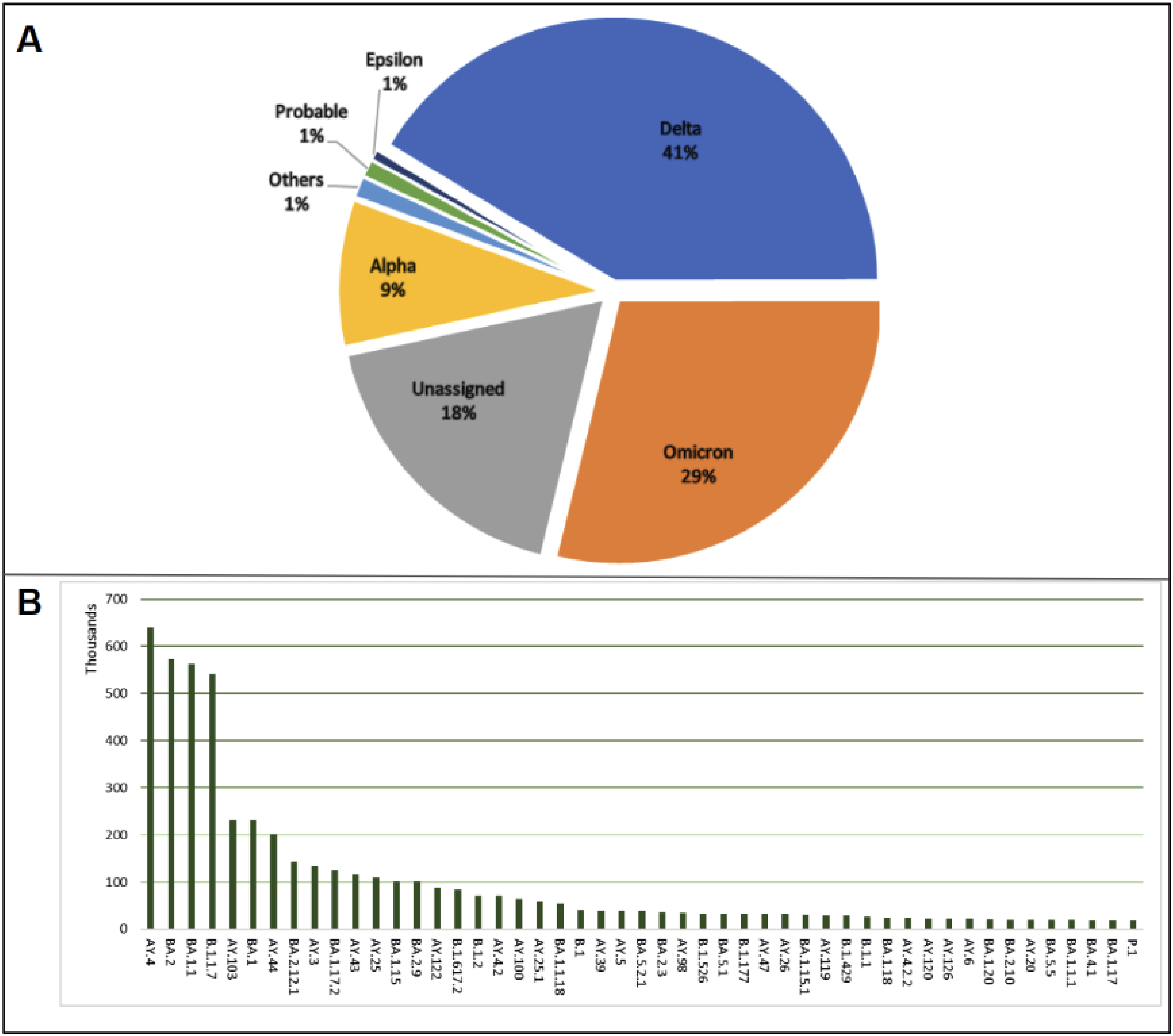
A
Epsilon 1%
Probable 1%
Others 1%
Delta 41%
Alpha 9%
Unassigned 18%
Omicron 29%
B
Thousands
700
600
500
400
300
200
100
0
AY.4
BA.2
BA.1.1
B.1.1.7
AY.103
BA.1
AY.44
BA.2.12.1
AY.3
BA.1.17.2
AY.43
AY.25
BA.1.15
BA.2.9
AY.122
B.1.617.2
B.1.2
AY.4.2
AY.100
AY.25.1
BA.1.1.18
B.1
AY.39
AY.5
BA.5.2.1
BA.2.3
AY.98
B.1.526
BA.5.1
B.1.177
AY.47
AY.26
BA.1.15.1
AY.119
B.1.429
B.1.1
BA.1.18
AY.4.2.2
AY.120
AY.126
AY.6
BA.1.20
BA.2.10
AY.20
BA.5.5
BA.1.1.1
BA.4.1
BA.1.17
P.1

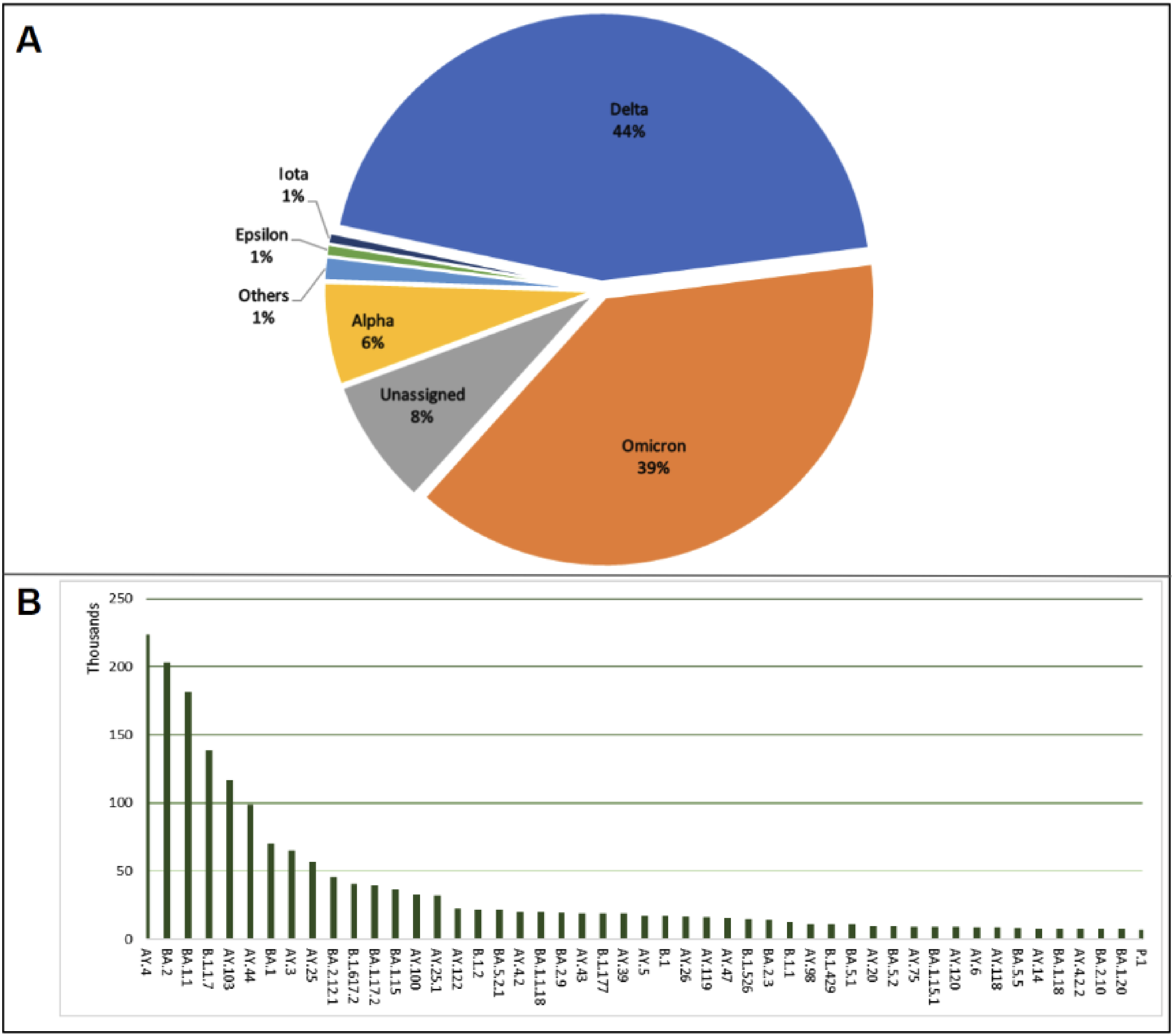
A
Delta
44%
Iota
1%
Epsilon
1%
Others
1%
Alpha
6%
Unassigned
8%
Omicron
39%
B
Thousands
250
200
150
100
50
0
AY.4
BA.2
BA.1.1
B.1.1.7
AY.103
AY.44
BA.1
AY.3
AY.25
BA.2.12.1
B.1.617.2
BA.1.17.2
BA.1.15
AY.100
AY.25.1
AY.122
B.1.2
BA.5.2.1
AY.4.2
BA.1.1.18
BA.2.9
AY.43
B.1.177
AY.39
AY.5
B.1
AY.26
AY.119
AY.47
B.1.526
BA.2.3
B.1.1
AY.98
B.1.429
BA.5.1
AY.20
BA.5.2
AY.75
BA.1.15.1
AY.120
AY.6
AY.118
BA.5.5
AY.14
BA.1.18
AY.4.2.2
BA.2.10
BA.1.20
P.1

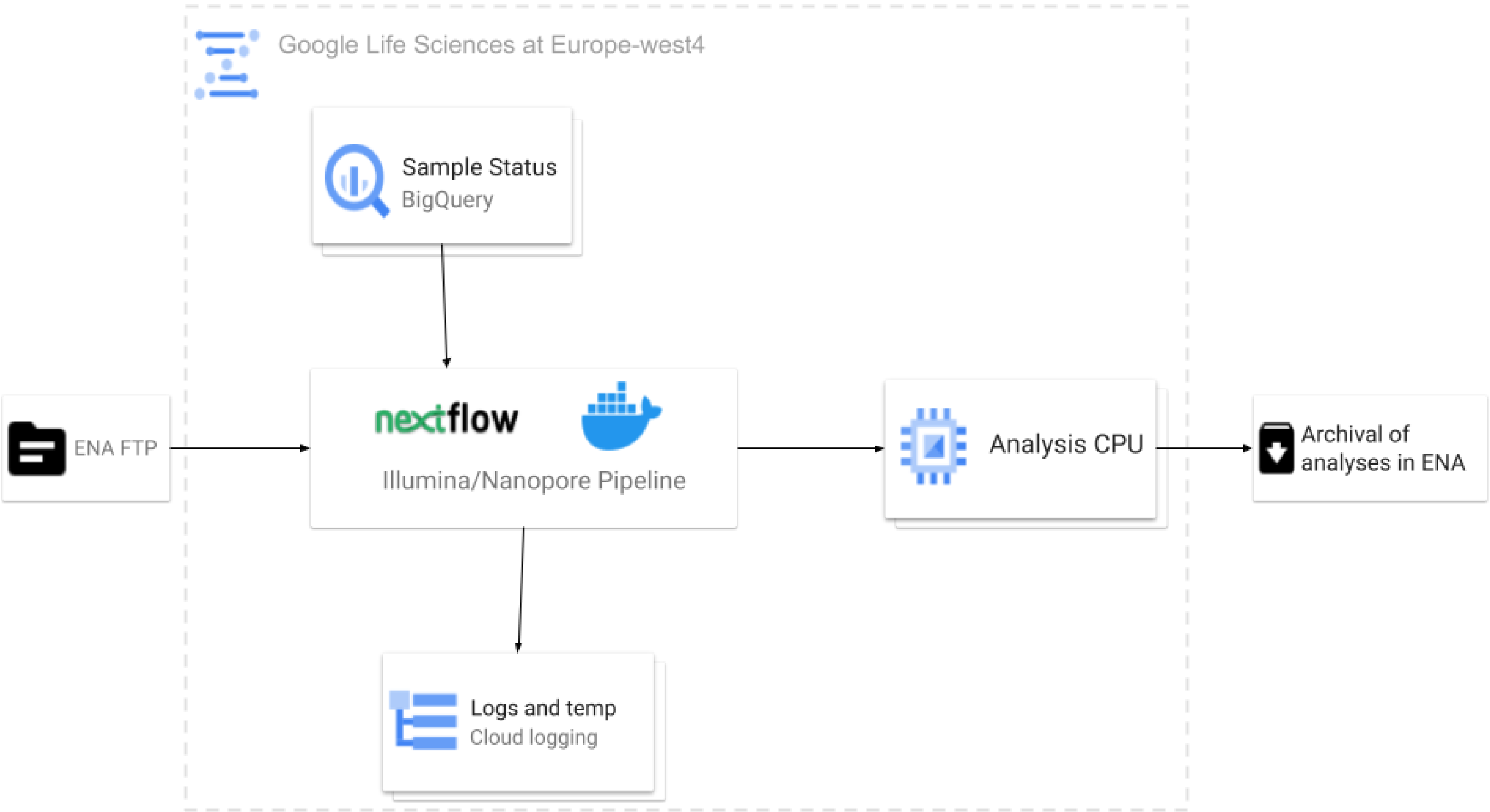
Google Life Sciences at Europe-west4
Sample Status
BigQuery
ENA FTP
nextflow
Illumina/Nanopore Pipeline
Analysis CPU
Archival of
analyses in ENA
Logs and temp
Cloud logging

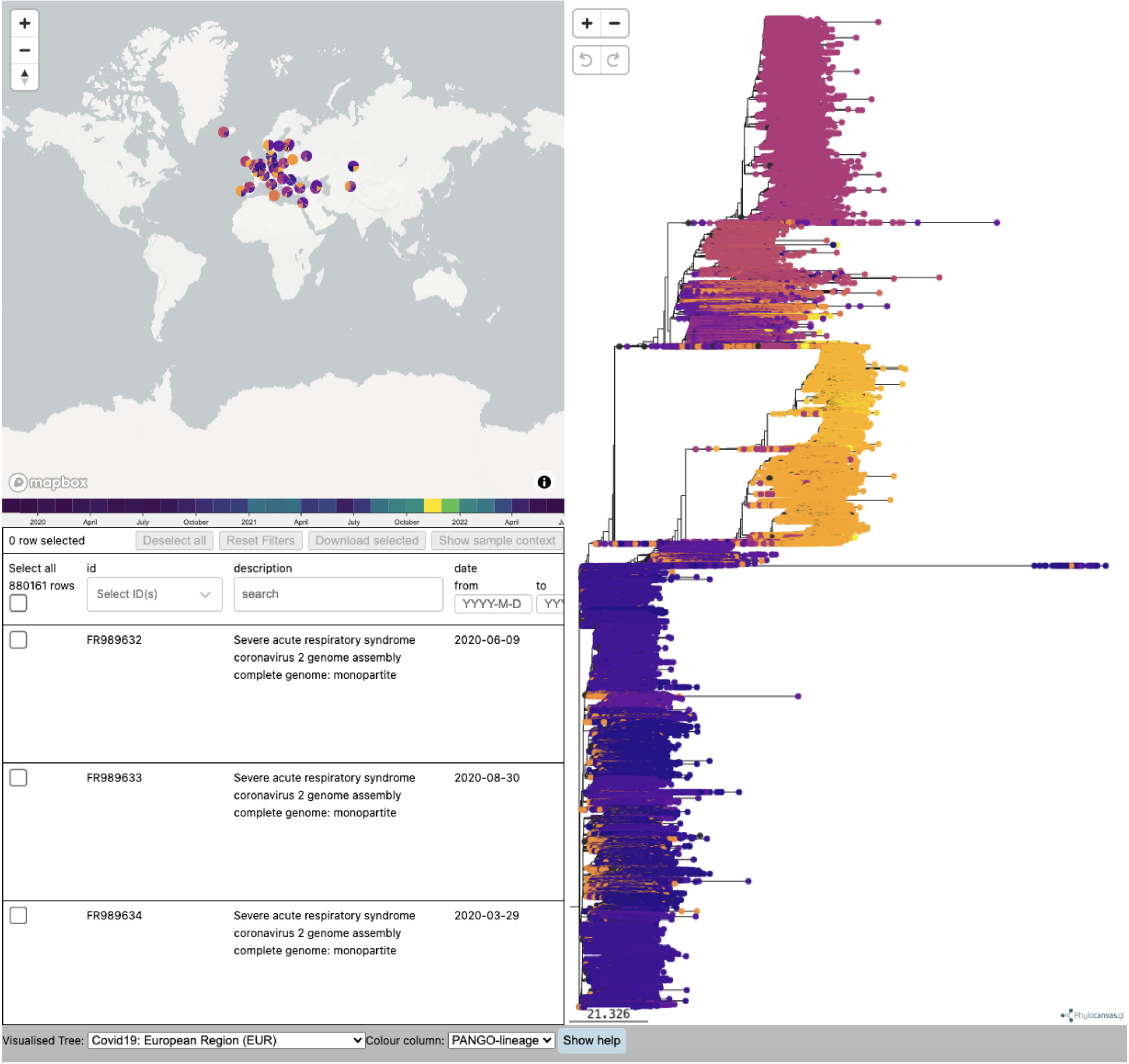

mapbox
2020
April
July
October
2021
April
July
October
2022
April
0 row selected
Deselect all
Reset Filters
Download selected
Show sample context
Select all
880161 rows
id
Select ID(s)
description
search
date
from
to
YYYY-M-D
FR989632
Severe acute respiratory syndrome coronavirus 2 genome assembly complete genome: monopartite
2020-06-09
FR989633
Severe acute respiratory syndrome coronavirus 2 genome assembly complete genome: monopartite
2020-08-30
FR989634
Severe acute respiratory syndrome coronavirus 2 genome assembly complete genome: monopartite
2020-03-29
21.326
Phylocanvas.gl
Visualised Tree:
Covid19: European Region (EUR)
Colour column:
PANGO-lineage
Show help

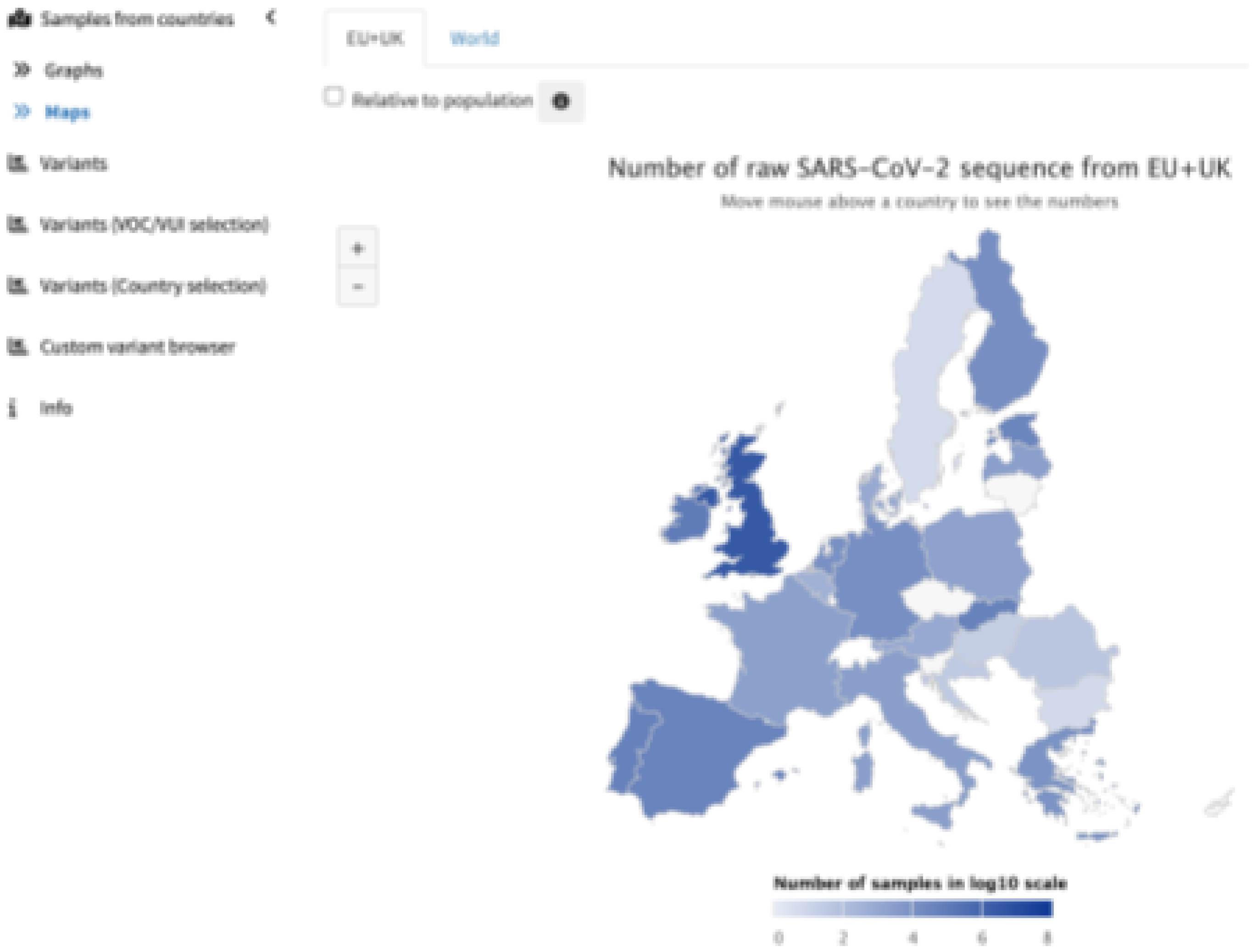

Samples from countries
Graphs
Maps
Variants
Variants (VOC/VUI selection)
Variants (Country selection)
Custom variant browser
Info
EU+UK
World
Relative to population
Number of raw SARS-CoV-2 sequence from EU+UK
Move mouse above a country to see the numbers
Number of samples in log10 scale
0
2
4
6
8


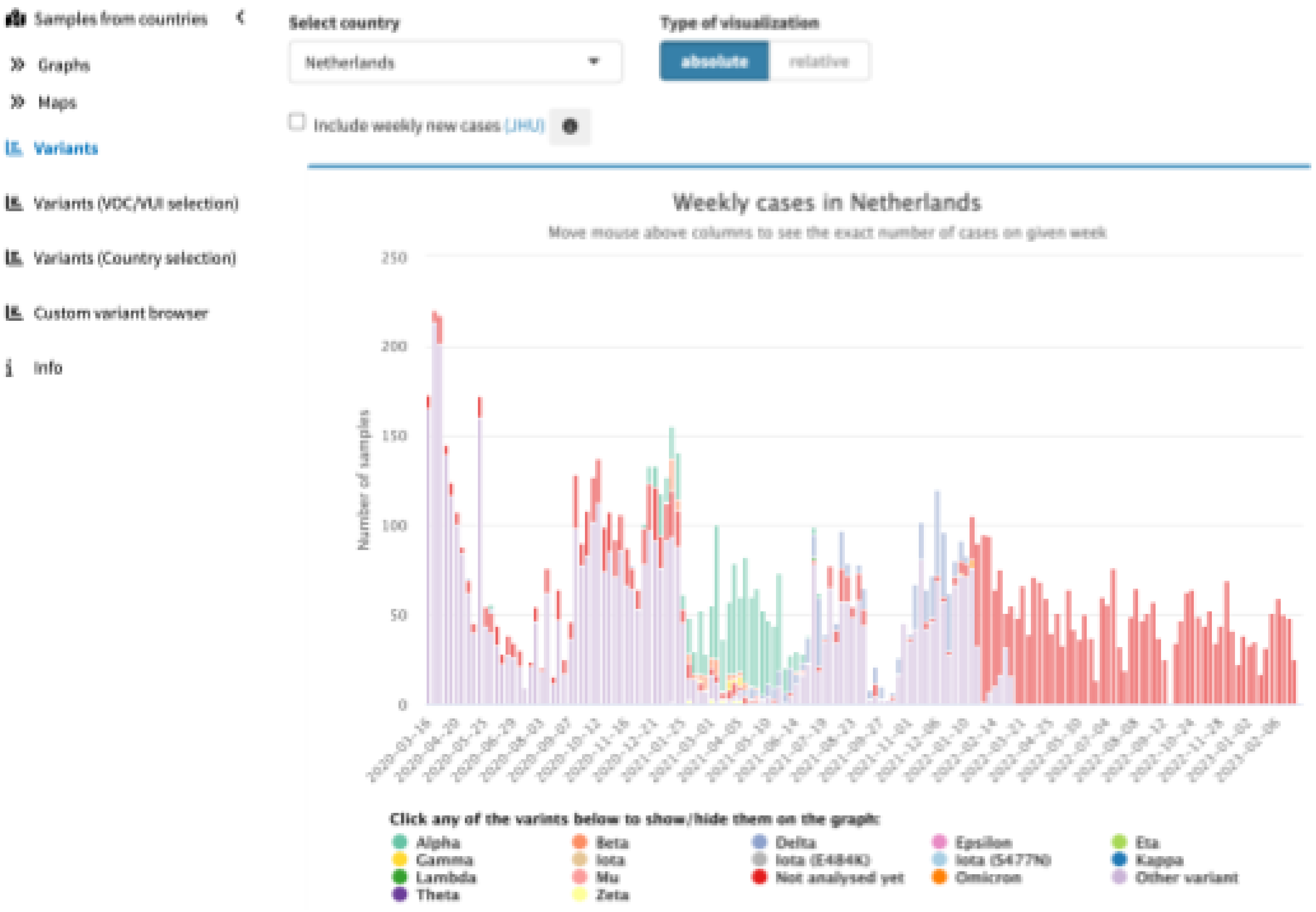

Samples from countries
Graphs
Maps
Variants
Variants (VOC/VUI selection)
Variants (Country selection)
Custom variant browser
Info
Select country
Netherlands
Type of visualization
absolute
relative
Include weekly new cases (JHU)
Weekly cases in Netherlands
Move mouse above columns to see the exact number of cases on given week
Number of samples
250
200
150
100
50
0
Click any of the varints below to show/hide them on the graph:
Alpha
Beta
Delta
Epsilon
Eta
Gamma
Iota
Iota (E484K)
Iota (S477N)
Kappa
Lambda
Mu
Not analysed yet
Omicron
Other variant
Theta
Zeta
